# Capturing functional two-dimensional nanosheets from sandwich-structure vermiculite for cancer theranostics

**DOI:** 10.1038/s41467-021-21436-5

**Published:** 2021-02-18

**Authors:** Xiaoyuan Ji, Lanlan Ge, Chuang Liu, Zhongmin Tang, Yufen Xiao, Wei Chen, Zhouyue Lei, Wei Gao, Sara Blake, Diba De, Bingyang Shi, Xiaobing Zeng, Na Kong, Xingcai Zhang, Wei Tao

**Affiliations:** 1 Center for Nanomedicine and Department of Anesthesiology, Brigham and Women’s Hospital, Harvard Medical School, Boston, MA USA; 2 Center Lab of Longhua Branch, Shenzhen People’s Hospital, Second Clinical Medical College of Jinan University, Shenzhen Guangdong, China; 3 Department of Infectious Disease, Shenzhen People’s Hospital, Second Clinical Medical College of Jinan University, Shenzhen Guangdong, China; 4 Integrated Chinese and Western Medicine Postdoctoral Research Station, Jinan University, Guangzhou Guangdong, China; 5 School of Engineering and Applied Sciences, Harvard University, Cambridge, MA USA; 6 Henan-Macquarie Uni Joint Centre for Biomedical Innovation, School of Life Sciences, Henan University, Kaifeng, Henan China; 7 Department of Biomedical Sciences, Faculty of Medicine and Health Sciences, Macquarie University, Sydney, NSW Australia; 8 Present Address: Academy of Medical Engineering and Translational Medicine, Medical College, Tianjin University, Tianjin, China

**Keywords:** Cancer therapy, Oncology, Nanoscale materials

## Abstract

Clay-based nanomaterials, especially 2:1 aluminosilicates such as vermiculite, biotite, and illite, have demonstrated great potential in various fields. However, their characteristic sandwiched structures and the lack of effective methods to exfoliate two-dimensional (2D) functional core layers (FCLs) greatly limit their future applications. Herein, we present a universal wet-chemical exfoliation method based on alkali etching that can intelligently “capture” the ultrathin and biocompatible FCLs (MgO and Fe_2_O_3_) sandwiched between two identical tetrahedral layers (SiO_2_ and Al_2_O_3_) from vermiculite. Without the sandwich structures that shielded their active sites, the obtained FCL nanosheets (NSs) exhibit a tunable and appropriate electron band structure (with the bandgap decreased from 2.0 eV to 1.4 eV), a conductive band that increased from −0.4 eV to −0.6 eV, and excellent light response characteristics. The great properties of 2D FCL NSs endow them with exciting potential in diverse applications including energy, photocatalysis, and biomedical engineering. This study specifically highlights their application in cancer theranostics as an example, potentially serving as a prelude to future extensive studies of 2D FCL NSs.

## Abstract (web summary)

Clay-based nanomaterials are of wide interest but problems extracting the 2D functional core layers have limited potential applications. Here, the authors report on the wet exfoliation of vermiculite by alkali etching to obtain the core layers and explore the application of the materials in cancer theranostics.

## Introduction

Layered materials whose two-dimensional (2D) lamellas are stacked to form three-dimensional structures via weak physical forces have played important roles throughout history^1–4^. The application of layered compounds can be traced back to 400 A.D., when Mayans used natural layered clays to make dyes^5^. Since then, scientific research involving layered materials has gradually evolved, culminating in the elucidation of the sheet structure of layered materials, detailed comprehension of their properties, and ultimately their delamination or exfoliation into atomically thin nanosheets, referred to as 2D nanomaterials^5–10^. Encouraged by the success of graphene, 2D nanomaterials with molecular or atomic thickness are emerging as functional materials and have attracted extensive attention because of their superlative properties^9–14^.

As a common building material and artistic medium of antiquity, clays, also referred to as phyllosilicate minerals, are fundamentally composed of tetrahedral silicon (SiO_2_) and/or aluminum oxide (Al_2_O_3_) crystal structures^15^. With the development and metamorphism seen in nature, a kind of clay such as vermiculite (VMT), biotite, flogopite, illite, etc., which belong to the 2:1 aluminosilicate family, have emerged. These kinds of clays consist of an octahedral layer of magnesium oxide (MgO) and ferric oxide (Fe_2_O_3_) sandwiched between two identical tetrahedral layers of SiO_2_ and Al_2_O_3_. The sandwiched layers are closely connected through water or metal ions, forming the three-dimensional structures of clay particles. Different exfoliation methods based on breaking the connection between the sandwiched layers, such as aqueous exfoliation^16^, ion and proteins-assisted aqueous exfoliation^17,18^, and organic polymer and epoxies-assisted organic solution exfoliation^19–21^, have been developed to prepare 2D clay nanosheets (NSs) with the sandwiched layer as a unit. Nevertheless, synthesizing high-quality sandwiched-layered NSs remains a huge challenge, even using complex procedures and imposing strict conditions.

Through an in-depth analysis of the physicochemical properties of the ingredients in each sandwiched layer, we found that the “bread layer” is composed of SiO_2_ and Al_2_O_3_, i.e., acidic and amphoteric oxides, which can be reacted with an alkali solution to generate corresponding acid radical ions. However, the functional core layers (FCLs) are composed of MgO and Fe_2_O_3_, alkaline oxides that are immune from etching by alkali. In addition, the “bread layer” seems to be of much smaller significance compared with the core layer in applications involving energy, catalysis, and biomedicine. Considering the previous studies in the silicon-based template removal^22,23^ and Lewis acidic etching for preparing MXenes^24^, it may be possible for a wet-chemical exfoliation method (based on alkali etching) to intelligently “capture” the ultrathin and biocompatible FCLs (MgO and Fe_2_O_3_) sandwiched between two identical tetrahedral layers (SiO_2_ and Al_2_O_3_) from VMT. Moreover, the main ingredients of FCL NSs are MgO and Fe_2_O_3_, both approved by the U.S. Food and Drug Administration (FDA) and widely used in the clinic. For example, OsteoCrete^®^, a product made with MgO, has been approved by the FDA for clinical bone repair. MgO is also widely used in the clinic and FDA-approved for the treatment of stomach diseases^25^. Fe_2_O_3_ nanoparticles have also been approved by the FDA for the treatment of iron deficiency^26^. It is worth mentioning that, the raw material VMT is also a commonly used traditional Chinese medicine, which is included in the Chinese pharmacopeia. Encouraged by the advantages of FCL NSs’ potential biocompatibility and our previous research experience with 2D nanomaterials in cancer field^10,27–31^, the exfoliation of FCL NSs and their applications in cancer theranostics are worth to be explored.

Here, we present a universal wet-chemical exfoliation method based on alkali etching that can intelligently “capture” the ultrathin and biocompatible FCLs (MgO and Fe_2_O_3_) sandwiched between two identical tetrahedral layers (SiO_2_ and Al_2_O_3_) from VMT. After PEGylation with amine-functionalized polyethylene glycol (PEG-NH_2_), the FCL-PEG NSs, with a bandgap of 1.4 eV and a conduction band at −0.6 eV, were able to produce ·O_2_^−^ from O_2_ under irradiation from a 658 nm laser, mediating photodynamic therapy (PDT). The Fe_2_O_3_ portion of the FCL NSs catalyzed Fenton reactions with H_2_O_2_ to generate ·OH, mediating chemodynamic therapy (CDT), an effect greatly enhanced by exposure to 658 or 808 nm lasers. In addition, the FCL-PEG NSs were capable of strongly regulating the tumor microenvironment (TME) by generating O_2_ and depleting glutathione (GSH), ameliorating the hypoxic and antioxidant capability of tumors. Meanwhile, for photothermal therapy (PTT), the FCL-PEG NSs exhibited high photothermal conversion efficiency when exposed to 808 nm laser irradiation, which leads to prominent synergistic and photo-enhanced PDT/CDT/PTT. The FCL-PEG NSs also showed good potential for photothermal, photoacoustic (PA), and fluorescence imaging. The obtained FCL-PEG NSs also showed good biocompatibility, indicating excellent potential in translational medicine. Therefore, this work not only provides a smart strategy to intelligently “capture” the sandwiched FCLs from clay materials, but also demonstrated proof-of-concept application of the obtained 2D NSs in cancer theranostics, which may also serve as a modest spur to induce future valuable contributions to other possible fields.

## Results

### Preparation and characterization of FCL-PEG NSs

**Fig. 1:**
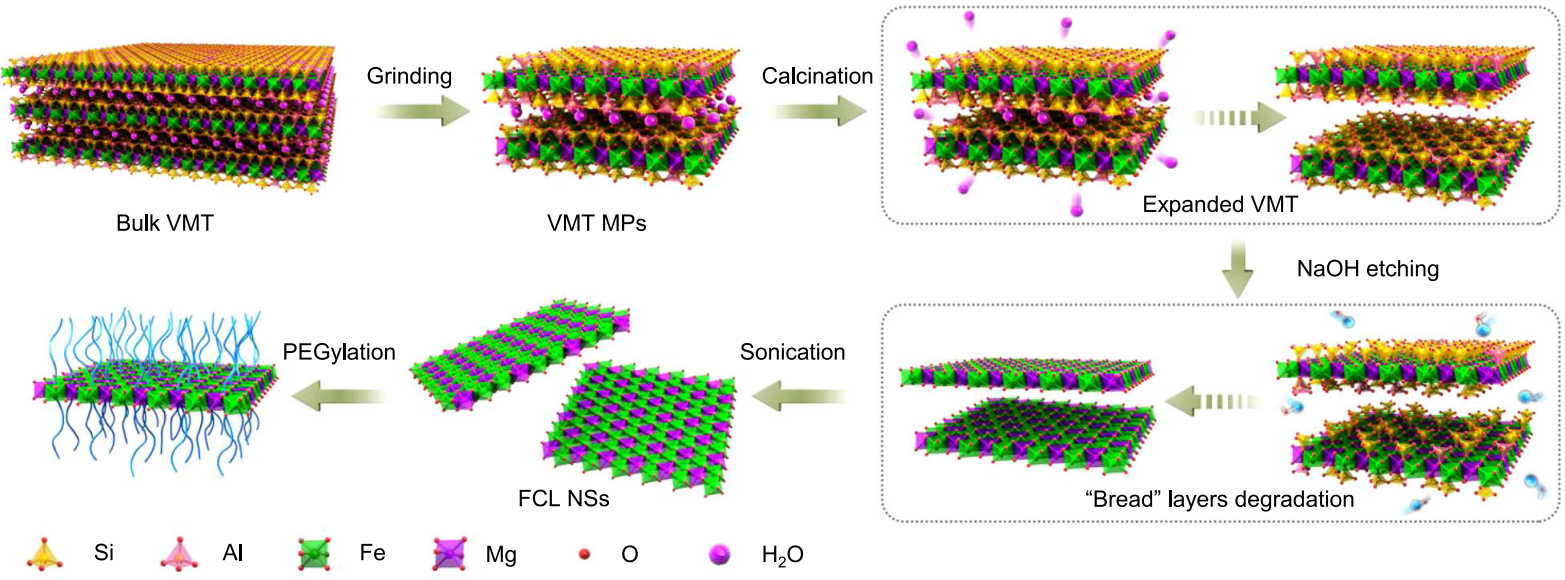
Schematic illustration of preparation processes of FCL-PEG NSs. The FCL NSs were prepared by coupling grinding, calcination, alkali etching, and liquid exfoliation. VMT vermiculite, MPs microparticles, FCL NSs functional core layers nanosheets.

**Fig. 2:**
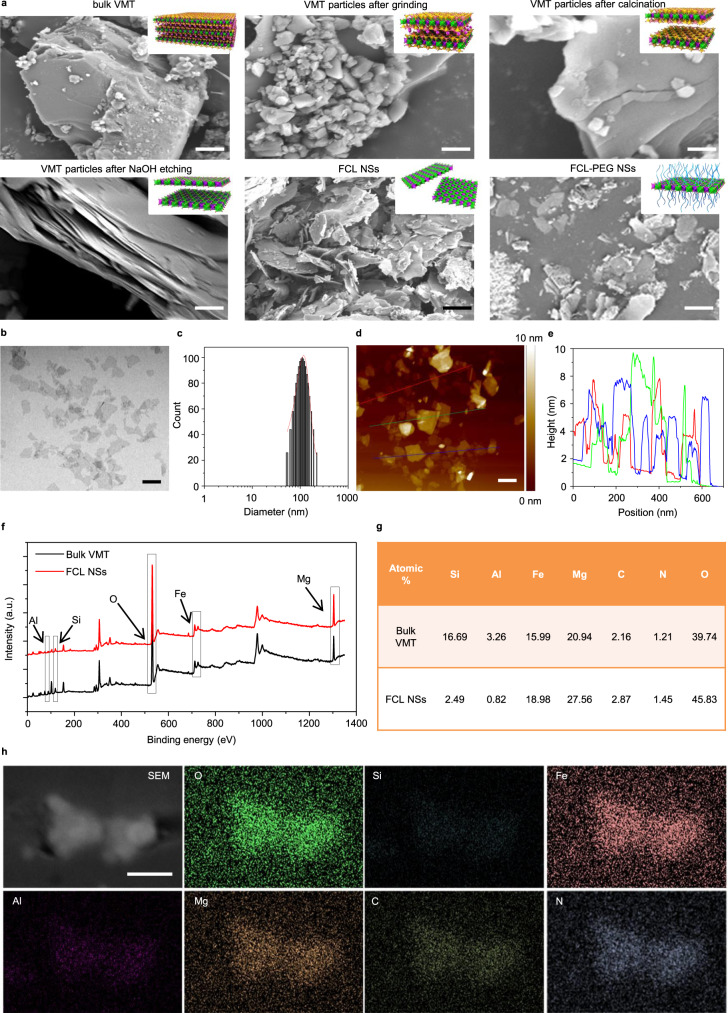
Characterization of FCL-PEG NSs. **a** SEM images of FCL-based NSs during their preparation: upper left—bulk VMT (scale bar 1 μm), upper middle—VMT particles after grinding (scale bar 1 μm), upper right—VMT particles after calcination (scale bar 100 nm), bottom left—VMT particles after NaOH etching (scale bar 100 nm), bottom middle—FCL NSs (scale bar 100 nm), and bottom right—FCL-PEG NSs (scale bar 100 nm). **b** TEM image (scale bar 100 nm), **c** size distribution, **d** AFM image (scale bar 100 nm), **e** thickness of FCL NSs, **f** XPS spectra, and **g** atomic percent of bulk VMT and FCL NSs, **h** SEM-EDX mapping images of FCL-PEG NSs (scale bar 100 nm for all panels). Three times each experiment was repeated independently with similar results in all these characterization figures. SEM scanning electron microscope, TEM transmission electron microscope, AFM atomic-force microscope, XPS X-ray photoelectron spectroscopy, EDX energy-dispersive X-ray detection.

In the first stage of this work, the ultrathin core layer of VMT (as an example) was successfully dissociated by a combination of ball-grinding, calcination, NaOH etching, and sonication (Fig. 1). First, relatively uniform VMT microparticles (MPs) (about 700 nm in size) were prepared through wet grinding in N-methyl pyrrolidone (NMP) at 111 × *g* for 30 min (Fig. 2a, from upper left to upper middle). Due to the strong interlayer forces resulting from the hydration layer between VMT layers, calcination at 800 °C was used to eliminate the hydration layer and achieve an expanded VMT with much larger interlamellar spacing (Fig. 2a, upper right). Then, the composition and physicochemical properties of the components of each sandwiched layer were analyzed and exhibited in Fig. 1. For each sandwiched layer of VMT, the “bread layers” were composed of SiO_2_ and Al_2_O_3_, which belong to acidic and amphoteric oxides; in contrast, the core layer is composed of MgO and Fe_2_O_3_, which are alkaline oxides. As is well known, acidic and amphoteric oxides (such as SiO_2_ and Al_2_O_3_, i.e., the “bread layers”) can easily be corroded by alkali solutions (such as NaOH and NaHCO_3_), generating the corresponding acid radical ions. Conversely, the alkaline oxides (such as MgO and Fe_2_O_3_, i.e., the core layers) are immune from alkali etching. Hence, etching with a hot alkaline solution (such as NaOH) was used to corrode the “bread” SiO_2_ and Al_2_O_3_ layers of VMT, which are very hard to biodegrade in TME, allowing the FCLs, composed of Fe_2_O_3_ and MgO, to be extracted (Fig. 2a, bottom left). Consequently, the interlamellar space became large enough for single-core layer exfoliation by simple probe sonication (Fig. 2a, bottom middle). As shown in Supplementary Figs. 1 and 2, after probe sonication, the average thickness and size of FCL NSs were 3.0 and 110 nm, respectively. To further confirm the corrosion of “bread” layers by alkaline solutions, the composition of bulk VMT and FCL NSs was analyzed using X-ray photoelectron spectroscopy (XPS). As shown in Fig. 2f and g, precisely because the NaOH etching only corroded the “bread layers” composed of SiO_2_ and Al_2_O_3_, the Si and Al content decreased significantly (from 16.69% and 3.26% to 2.49% and 0.82%, respectively), accompanied by a negligible change in Fe and Mg content, further demonstrating the disintegration of the “bread layers”. In addition, X-ray energy spectrum (EDX) mapping images provide stronger evidence for the disintegration of the “bread” layers, in which the color of Si and Al in FCL NSs faded considerably compared with those of bulk VMT (Fig. 2h and Supplementary Fig. 3). For nanomedicines used in vivo, physiological stability and dispersibility are important indicators that the FCL NSs were further modified by positively charged PEG-NH_2_ (Fig. 2a, bottom right). As shown in Supplementary Fig. 4, FCL-PEG NSs showed negligible agglomeration after 24-h incubation, and remarkably better stability and dispersibility in phosphate-buffered saline (PBS), fetal bovine serum (FBS) solution, and Dulbecco’s modified Eagle medium (DMEM) compared to bare FCL NSs. As shown in Fig. 2b–e, the average thickness of FCL-PEG NSs increased to 6 nm, demonstrating successful PEG-NH_2_ functionalization. The average size of FCL-PEG NSs decreased to 105 nm, due to the use of bath sonication to break down FCL NSs during PEGylation^31,32^. To further confirm the PEGylation of FCL NSs, Fourier-transform infrared (FTIR) spectra were applied to test the composition of FCL-PEG NSs (Supplementary Fig. 5). The characteristic absorption peaks at about 1000 and 2900 cm^−1^ resulted from the stretching vibrations of -C–O-C- and -CH in PEG-NH_2_ (respectively), confirming the success of PEG-NH_2_ functionalization. Moreover, the elements of PEG-NH_2_, including O, N, and C, and the elements of FCL NSs, including Mg and Fe, were all detected in EDX mapping of FCL-PEG NSs (Fig. 2h), further demonstrating the successful PEGylation of FCL NSs.

### TME regulation through FCL-PEG NSs

O_2_ concentration and redox homeostasis in TME play important roles in the metabolism of cells^33–37^. The strong oxidizability of Fe^3+^ could catalyze the oxidation of GSH and H_2_O_2_ to generate GSSG and O_2_ through the following formulas^18^.

1 $${\mathrm{Fe}}^{3 + } + {\mathrm{GSH}} \to {\mathrm{Fe}}^{2 + } + {\mathrm{GSSG}}$$

2 $${\mathrm{Fe}}^{3 + } + {\mathrm{H}}_2{\mathrm{O}}_2 \to {\mathrm{Fe}}^{2 + } + {\mathrm{H}}_2{\mathrm{O}} + {\mathrm{O}}_2 \uparrow$$

**Fig. 3:**
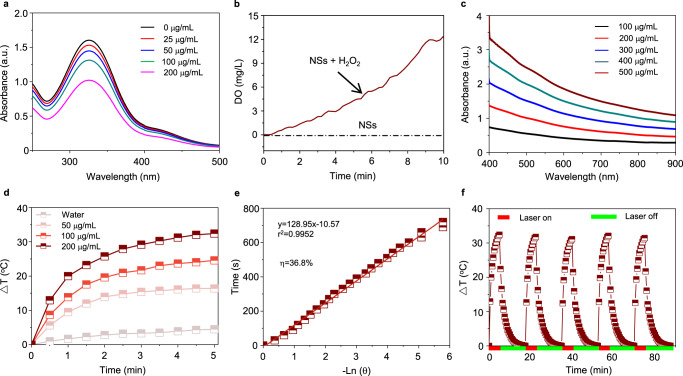
TME modulation and photothermal conversion performance of FCL-PEG NSs. **a** GSH degradation profile treated with FCL-PEG NSs at different concentrations. **b** O_2_ production in H_2_O_2_ solution treated with FCL-PEG NSs. **c** The light absorbance spectra of FCL-PEG NSs. **d** The photothermal conversion performance of FCL-PEG NSs (0–200 μg/mL) irradiated by an 808 nm laser (2 W/cm^2^). **e** The linear relationship between −ln *θ* and time. **f** Heating and cooling curves of the FCL-PEG NSs. GSH glutathione.

In this set of experiments, the capacity of FCL-PEG NSs to regulate the TME, including producing O_2_ and consuming GSH, was evaluated. As observed in the XPS spectra of Fe 2p (Supplementary Fig. 6), there was abundant Fe^3+^ present in prepared FCL-PEG NSs, which could react with GSH and H_2_O_2_ to reduce antioxidant capability and increase the O_2_ content of tumors via a series of redox reactions. Figure 3a illustrates fast and concentration-dependent GSH depletion, which could reduce antioxidant capability and lead to a quick accumulation of ROS. Moreover, as shown in Fig. 3b, continuous O_2_ production could be attributed to the redox reaction between Fe^3+^ and H_2_O_2_, providing the substrate for PDT. Hence, FCL-PEG NSs have strong potential to facilitate ROS-mediated therapies through modulating TME, which includes consumption of GSH and production of O_2_.

### Photothermal conversion of FCL-PEG NSs

After confirming the good performance of FCL-PEG NSs in regulating TME, the photothermal conversion properties of FCL-PEG NSs were evaluated. The broad, strong light absorption of FCL-PEG NSs ranging from UV to NIR is presented in Fig. 3c and Supplementary Figs. 7 and 8. For PTT, two basic principles of laser irradiation need to be satisfied to complete effective tumor ablation: minimizing tissue scattering and absorption to achieve effective light penetration and minimize tissue self-heating, as well as high photothermal conversion efficiency of photothermal agents. As reported by others^38–40^ near-infrared (NIR) light, especially 808 nm laser, has the highest equilibrium point among tissue penetration, tissue self-heating, and photothermal conversion of photothermal agents, which is the main reason that 808 nm laser is preferred for clinical photothermal therapy applications. Hence, to assess the photothermal conversion efficiency (*η*) of FCL-PEG NSs, FCL-PEG NSs aqueous solutions of different concentrations were irradiated by an 808 nm laser with different powers. To guarantee the accuracy of temperature changes, both an infrared radiation (IR) thermal camera and a traditional liquid thermometer were employed to detect the photothermal conversion of FCL-PEG NSs. As shown in Fig. 3d and Supplementary Fig. 9 (recorded by an IR thermal camera and verified by a liquid thermometer), rapid laser power and concentration-dependent temperature increase of FCL-PEG NSs were noted. Specifically, the highest temperature increment (Δ*T*_max_) of 32.4 °C was obtained with FCL-PEG NSs concentration of 0.2 mg/mL at a power intensity of 2 W/cm^2^. Calculated using a series formula included in the experimental section hereof, the *η* of FCL-PEG NSs was determined to be 36.8% (Fig. 3e and Supplementary Fig. 10), higher than most conventional photothermal agents. It is well known that photothermal stability is another key evaluation criterion for a good photothermal agent. A heating and cooling cycles experiment was conducted to assess the photothermal stability of FCL-PEG NSs. As demonstrated in Fig. 3f, a rapid temperature increase was observed when the FCL-PEG NSs aqueous solutions were exposed to an 808 nm laser. After the laser was turned off, the temperature of the FCL-PEG NSs solution gradually lowered to room temperature due to the temperature difference. Negligible changes were noted in photothermal conversion performance during 5 heating and cooling cycles, demonstrating the high photostability of FCL-PEG NSs.

### FCL-PEG NSs-mediated reactive oxygen species (ROS) generation

**Fig. 4:**
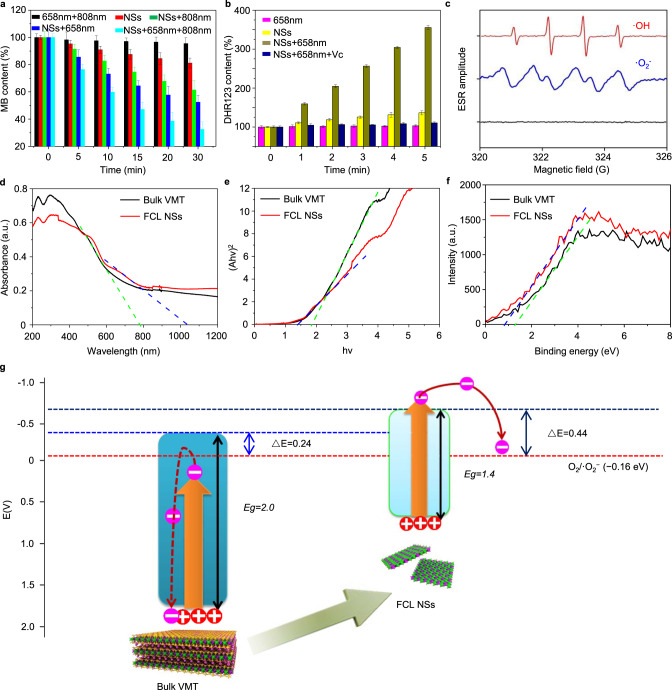
ROS generation and mechanism of FCL-PEG NSs. **a** The CDT effect mediated by FCL-PEG NSs using MB as the probe. The data show mean ± s.d., *n* = 3. **b** The PDT effect mediated by FCL-PEG NSs using DHR123 as the probe. The data show mean ± s.d., *n* = 3. **c** The signal of ROS generated by FCL-PEG NSs in EPR spectra. **d** All-solid-state UV–vis-NIR absorbance spectra of bulk VMT and FCL-PEG NSs. **e** The bandgap of bulk VMT and FCL-PEG NSs calculated by Kubelka–Munk equation. **f** CB of bulk VMT and FCL-PEG NSs. **g** Schematic illustration of photo-excited electron-hole separation and transfer mechanism of bulk VMT and FCL NSs. CDT chemodynamic therapy, MB methylene blue, PDT photodynamic therapy, DHR123 dihydrorhodamine 123, EPR electron paramagnetic resonance, UV–vis-NIR ultraviolet-visible-near-infrared red, CB conductive band.

The capacity of FCL-PEG NSs for the production of ROS, such as ·OH and ·O_2_^−^, and its potential as an excellent photosensitizer for CDT and PDT, were assessed^41–44^. Initially, methylene blue (MB), a specific indicator for ·OH, was used to test the capacity of FCL-PEG NSs for generating ·OH and mediating CDT. In only the laser (650 and 808 nm) and H_2_O_2_ group, no obvious degradation of MB was observed (Fig. 4a and Supplementary Fig. 11a). In comparison, due to the disproportionate reactions of H_2_O_2_ catalyzed FCL-PEG NSs, an evident degradation of MB (18.8%) caused by the generation of ·OH was shown (Fig. 4a and Supplementary Fig. 11b). More interestingly, much higher degradation of MB, resulting from the improved FCL-PEG NSs-mediated Fenton reaction, was observed when the reaction system was exposed to 808 and 658 nm laser irradiation, which reached 38.5% (Fig. 4a and Supplementary Fig. 11c) and 47.5% (Fig. 4a and Supplementary Fig. 11d), respectively. To explain the enhanced ·OH generation by 808 nm laser irradiation, the photothermal conversion-caused temperature increase might promote ionization of FCL-PEG NSs, giving rise to improved Fenton reaction efficacy. The primary reason for the enhanced ·OH generation by 658 nm laser irradiation might be the ·OH generation from OH^−^ catalyzed by the photo-generated holes in the valence band (VB) of FCL-PEG NSs. Hence, the greatest degradation of MB (67.5%) was achieved by exposing the reaction system to 808 and 658 nm laser irradiation, further enhancing both the Fenton reaction and ·OH generation. Although MB is a type of photosensitizer that can be used for PDT under light irradiation with a wavelength 600–670 nm, the ^1^O_2_ generated by MB under irradiation by the 658 nm laser does not affect the degradation of MB, which was demonstrated in the 658 + 808 nm group here and by previous research^45,46^. Moreover, for a more precise representation of ·OH generation by FCL-PEG NSs and enhanced by 808 and 658 nm lasers, another indicator for ·OH (3,3′,5,5′-tetramethylbenzidine/TMB) was also applied. As shown in Supplementary Fig. 12, obvious ·OH generation was observed when FCL-PEG NSs were added into the H_2_O_2_ solution. Besides, enhanced ·OH generation under 808 and 658 nm laser irradiation are also shown in Supplementary Fig. 12, further demonstrating the strong potential of FCL-PEG NSs-mediated CDT for cancer treatment.

Next, the capacity of PDT mediated by FCL-PEG NSs through generating ·O_2_^−^ was tested and evaluated via using dihydrorhodamine 123 (DHR123) as the specific indicator of ·O_2_^−^. As shown in Fig. 4b and Supplementary Fig. 13a, a negligible increase in DHR123 fluorescence was observed under exposure to 658 nm laser irradiation for 1 h, indicating the good stability of DHR123 treated with 658 nm laser irradiation. Introducing oxygen vacancy onto the surface of metallic oxides has been demonstrated to efficiently generate ·O_2_^−^ from O_2_. After calcination, NaOH etching, and sonication, oxygen vacancies on the surface of FCL NSs were tested by electron paramagnetic resonance (EPR) spectra and XPS analysis. As shown in Supplementary Fig. 14, a slight EPR signal should originate from the oxygen vacancy-trapped electrons, which means that some oxygen vacancies were generated on the surface of FCL NSs^47^ Because two extra electrons are left once an oxygen atom is removed from the surface of the metallic oxide, that will lead to increased electron cloud density around the metal and O atoms near the oxygen vacancy sites^48^. Comparison of high-resolution XPS spectra in the Mg 1s (Supplementary Fig. 15a) and O 1s (Supplementary Fig. 15b) regions indicates that the introduction of oxygen vacancy into FCL NSs results in decreased binding energy for both Mg and O, which not only confirm the generation of oxygen vacancy, but also demonstrate the oxygen vacancy mainly derived from MgO. Besides, the weak EPR signal and slightly decreased binding energies, there were very few oxygen vacancies on the surface of FCL NSs. Due to the presence of oxygen vacancies on the surface of FCL-PEG NSs, which could catalyze ·O_2_^−^ generation from O_2_, a mild fluorescence increase of DHR123 is shown in Fig. 4b and Supplementary Fig. 13b, also confirming the limited oxygen vacancies. By contrast, the fluorescence intensity of DHR123 increased over threefold after being exposed to 658 nm laser irradiation (Fig. 4b and Supplementary Fig. 13c), which might be attributed to photo-generated electrons in the conduction band (CB) of FCL-PEG NSs, which have a strong ability to catalyze the reduction of O_2_. In consideration of the short lifetime and high chemical activity of ·OH and ·O_2_^−^, the very reliable technique of electron paramagnetic resonance (EPR) was applied to further detect the species of ROS. Both ROS species (·OH and ·O_2_^−^) showed a significant EPR signal, further indicating the strong ROS-generating capability of FCL-PEG NSs (Fig. 4c).

### Mechanistic understanding of enhanced ROS generation by FCL-PEG NSs

For further identification of ROS species and explanation of the mechanism of enhanced ROS generation, XPS spectra, and solid UV–vis-NIR absorption spectra were applied to determine the electronic band structure of FCL-PEG NSs. As shown in the solid UV–vis-NIR absorption spectra of FCL-PEG NSs (Fig. 4d), bulk VMT and prepared FCL-PEG NSs both had light absorption throughout the whole visible and NIR region. Moreover, prepared FCL-PEG NSs possessed a broader absorption threshold than did VMT, which means that FCL-PEG NSs have a stronger light absorption ability and relatively narrow bandgap (E_g_). The E_g_ of bulk VMT and FCL-PEG NSs were calculated to be 2.0 and 1.4 eV, respectively, based on the Kubelka–Munk conversion (Fig. 4e)^49^. The VBs of bulk VMT and FCL-PEG NSs were determined to be 1.6 and 0.8 eV, respectively, from the XPS spectra (Fig. 4f). So, the CBs of bulk VMT and FCL-PEG NSs were calculated to be −0.4 and −0.6 eV, the differences between the corresponding E_g_s and VBs^50,51^. Therefore, as schematically illustrated in Supplementary Fig. 16, efficient photo-excited electrons and holes separation and ·O_2_^−^ generation from O_2_ in CB of FCL-PEG NSs were achieved. To further analyze the influence of the tunable electron band structure on the efficiency of the photodynamic effect of FCL-PEG NSs, the mechanism of photo-induced electron-hole pairs separation and transfer was schematically illustrated in much greater detail and shown in Fig. 4g. Under irradiation by a certain wavelength laser and certain light energy, the electron-hole pairs in the VB of FCL-PEG NSs with a small E_g_ of 1.4 eV were easily excited and separated, with electron transfer to the CB of FCL-PEG NSs. However, for the bulk VMT with a large E_g_ of 2.0 eV, electron-hole pair separation and transfer to CB need much more light energy. As shown in Fig. 4g, given insufficient light energy irradiation, the electrons might retreat and recombine with the holes in the VB. Moreover, even if the light energy is sufficient for bulk VMT with a large E_g_, the CB edges remain another crucial factor for efficient ROS generation. Considering that the reduction potential (E^0^) of O_2_/·O_2_^−^ is −0.16 eV, there is a match of energy bands among bulk VMT and FCL-PEG NSs with the reduction potential of O_2_/·O_2_^−^. Furthermore, the efficacy of electron transfer could be affected by the relative energy position (ΔE), in which the bigger ΔE guarantees the higher electron transfer efficacy. As shown in Fig. 4g, the ΔE between the CB of FCL-PEG NSs and E^0^ of O_2_/·O_2_^−^ was much bigger than that with the CB of bulk VMT. Hence, after capture from the sandwiched structure of VMT, the bioactive layers FCL-PEG NSs with a much more appropriate electron band structure exhibited a much stronger photodynamic effect with more ROS generation. This tunable electron band structure obtained via this smart strategy might also be suitable for other 2:1 aluminosilicates, which would provide much more excellent photosensitizers and photocatalysts for photonic therapy, energy conversion, and so on.

### In vitro antitumor strategy mediated by FCL-PEG NSs

**Fig. 5:**
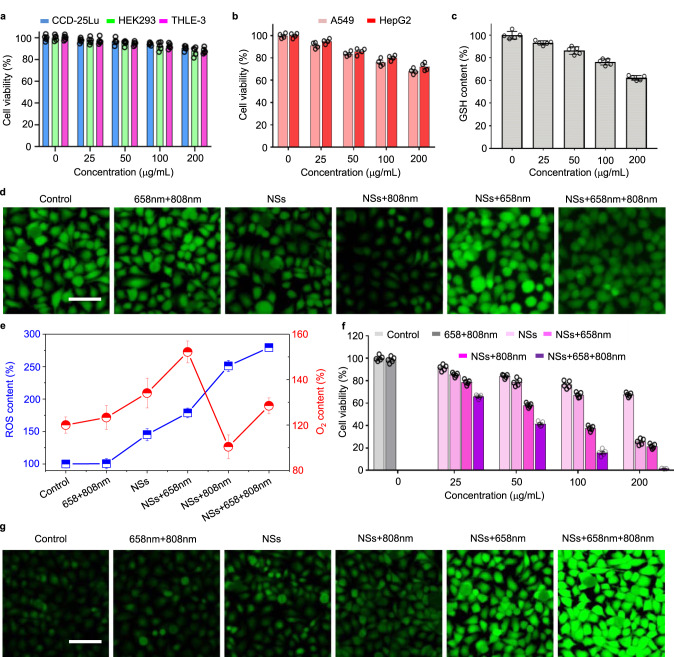
In vitro photo-enhanced chemodynamic, photodynamic, and photothermal therapy. **a** Normal (CCD-25Lu, HEK 293, and THLE-3) and **b** cancer cells (A549 and HepG2) viability treated with FCL-PEG NSs at different concentrations for 24 h. The data show mean ± s.d., *n* = 5 biologically independent cells. **c** GSH content of HepG2 cells treated with FCL-PEG NSs at different concentrations for 24 h. The data show mean ± s.d., *n* = 5 biologically independent cells. **d** CLSM images of O_2_ evolution of HepG2 cells treated with FCL-PEG NSs with or without 658 and 808 nm laser irradiation. Three times each experiment was repeated independently with similar results. **e** ROS and O_2_ content of HepG2 cells under different treatments. The data show mean ± s.d., *n* = 3 biologically independent cells. **f** CLSM images of ROS generation treated with FCL-PEG NSs with or without 658 and 808 nm laser irradiation. Three times each experiment was repeated independently with similar results in all these figures. **g** Antitumor effect of FCL-PEG NSs at different concentrations. The data show mean ± s.d., *n* = 5 biologically independent cells. Scale bars, 50 μm for all panels.

In the next set of experiments, the TME-modulating and ROS-generating capacity, as well as the antitumor effect in vitro of FCL-PEG NSs were further assessed. First, the biocompatibility of FCL-PEG NSs with normal and cancer cells, such as human lung fibroblasts (CCD-25Lu), human embryonic kidney cells (HEK 293), human liver epithelial cells (THLE-3), human hepatoma carcinoma cells (HepG2), and human lung cancer cells (A549), were tested via MTT assay. As shown in Fig. 5a and b, the FCL-PEG NSs exhibited evident specific cytotoxicity to cancer cells and good biocompatibility with normal cells. Although normal cells have more oxygen than cancer cells, which might make it much easier to generate ·O_2_^−^ from O_2_ catalyzed by the oxygen vacancy on the surface of FCL-PEG NSs, the limited oxygen vacancy on the surface of FCL-PEG NSs and short lifetime of ·O_2_^−^ guarantee good biocompatibility with normal cells. Hence, the main reason for the specific cytotoxicity to cancer cells of FCL-PEG NSs may be that the high content of H_2_O_2_ in TME facilitated the Fenton reaction of FCL-PEG NSs and generated more cytotoxic ·OH. Next, for the TME-modulating capacity of FCL-PEG NSs, the O_2_-generating and GSH-consuming performance in HepG2 cells was tested by using an O_2_ probe [Ru(dpp)_3_]Cl_2_ (RDPP) and a GSH assay kit. As shown in Fig. 5c, a rapid and FCL-PEG NSs concentration-dependent GSH-consuming performance was observed, which can be ascribed to the reaction between Fe^3+^ in FCL-PEG NSs and GSH. The O_2_ evolution via Fenton reaction of FCL-PEG NSs with H_2_O_2_ in TME is shown in Fig. 5d and e. Compared with the control and lasers-only groups, O_2_ generation was clearly shown in cells treated with FCL-PEG NSs, with a drastic decrease in green fluorescence. As demonstrated in Fig. 4a, 808 nm laser irradiation caused photothermal conversion and enhanced the Fenton reaction, so that much O_2_ generation was shown in Fig. 5d treated with FCL-PEG NSs and irradiated by an 808 nm laser. Next, 2,7-dichlorofluorescein diacetate (DCFH-DA) was employed as a probe to test the intracellular ROS content of FCL-PEG NSs in HepG2. As shown in Fig. 5e and f, the control group and the group treated with lasers only exhibited negligible ROS signal. A slightly stronger green fluorescence signal was observed in cells treated with FCL-PEG NSs, which might be mainly ascribed to ·OH generation via Fenton reaction with intracellular H_2_O_2_. As confirmed above, 808 nm laser irradiation-caused photothermal conversion likely enhanced the Fenton reaction, and the ·OH generation via Fenton reaction was improved, indicated by a stronger green fluorescence signal of ROS. Moreover, much stronger fluorescence emission was exhibited by cells treated with FCL-PEG NSs and exposed to 658 nm laser irradiation, further demonstrating enhanced Fenton reaction and laser-mediated photodynamic effect. Then, the antitumor effect of FCL-PEG NSs was tested in vitro using HepG2 and A549 as model cancer cells. An obvious dose-dependent antitumor effect of FCL-PEG NSs was observed after treatment with 658 or 808 nm laser irradiation (Fig. 5g and Supplementary Fig. 17). This suggested the FCL-PEG NSs could act as an efficient photosensitizer or photothermal agent for cancer PDT or PTT. Furthermore, the cells treated with FCL-PEG NSs and irradiated by 658 and 808 nm lasers were nearly all dead, demonstrating the strong potential of FCL-PEG NSs for effective photo-enhanced PDT/CDT/PTT.

### In vivo antitumor strategy mediated by FCL-PEG NSs

**Fig. 6:**
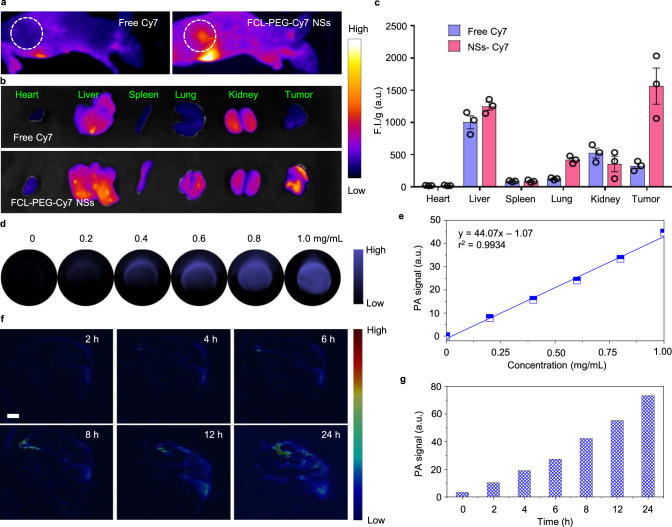
In vivo imaging and biodistribution study. **a**, **b** FCL-PEG NSs-mediated fluorescence images of tumor-bearing mice. **c** Semi-quantitative biodistribution of FCL-PEG-Cy7 NSs. The data show mean ± s.d., *n* = 3 biologically independent mice. **d**, **e** PA images and values of FCL-PEG NSs at different concentrations. **f**, **g** PA images and semi-quantitative analysis of PA values of the tumor site. The color gradient is arbitrary and does not have units in these figures.

**Fig. 7:**
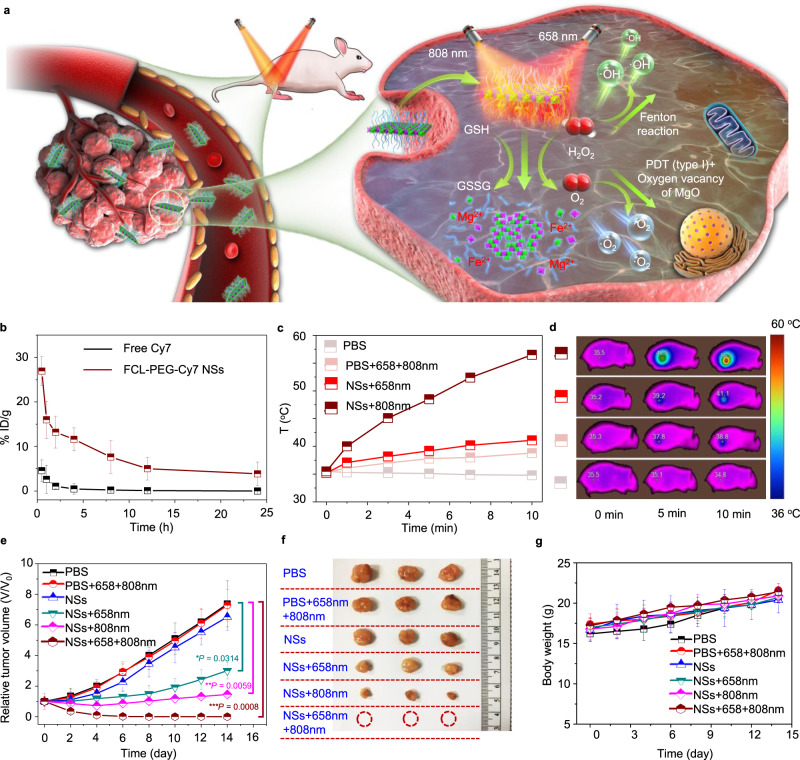
In vivo photo-enhanced chemodynamic, photodynamic, and photothermal therapy. **a** Schematic illustration of photonic therapy based on FCL-PEG NSs. **b** Blood circulation performance of FCL-PEG-Cy7 NSs and free Cy7. The data show mean ± s.d., *n* = 3 biologically independent animals. **c** Photothermal heating curves and **d** infrared thermographic images of tumor-bearing nude mice. **e** Antitumor effects of FCL-PEG NSs combined with different treatments. The data show mean ± s.d., *n* = 5 biologically independent mice, and significance was determined using a two-tailed *t*-test (*P < 0.05, **P < 0.01, ***P < 0.001). **f** Tumor images of different treatments. **g** Body weight of mice under different treatments. The data show mean ± s.d., *n* = 5 biologically independent mice.

Encouraged by the strong potential of FCL-PEG NSs in vitro, their performance as photosensitizers and antitumor photothermal agents in vivo was investigated. Based on the HepG2 xenograft-tumor model established on the backs of nude mice, the biodistribution of FCL-PEG NSs was investigated using FCL-PEG-Cy7 NSs as a fluorescent imaging agent. As shown in Supplementary Fig. 18, an obvious characteristic absorption peak of Cy7 was exhibited in the absorbance spectrum of FCL-PEG-Cy7 NSs, which signified the successful functionalization of FCL-PEG-Cy7 NSs. Abundant accumulation of FCL-PEG-Cy7 NSs at tumor sites is shown in Fig. 6a after 24 h intravenous (i.v.) injection. By contrast, almost no accumulation of free Cy7 can be observed in tumor sites at the same time point. The biodistribution of FCL-PEG-Cy7 NSs in the main organs and tumors 24 h post-injection are shown in Fig. 6b, calculated and listed in Fig. 6c. High accumulation of FCL-PEG-Cy7 NSs in the tumor indicated the potential of FCL-PEG NSs in cancer therapy. The fluorescence of FCL-PEG-Cy7 NSs in blood after different intervals, shown in Fig. 7b, demonstrated that FCL-PEG-Cy7 NSs possessed longer circulation time than free Cy7. Next, the potential of FCL-PEG NSs as PA imaging agents were also evaluated both in vitro and in vivo. Figure 6d and e shows a strong and concentration-dependent PA signal at 800 nm, supporting the promising potential of FCL-PEG NSs, as PA imaging agents^52^. Hence, PA imaging in the HepG2 xenograft-tumor model using i.v.-injected FCL-PEG NSs as PA imaging agents was performed. After different time intervals (2, 4, 6, 8, 12, and 24 h), PA signals of FCL-PEG NSs at the tumor site were recorded (Fig. 6f), reflecting a high accumulation of FCL-PEG NSs at the tumor site. Tumor-site accumulation of FCL-PEG NSs was also quantitatively analyzed and shown in Fig. 6g, suggesting the potential of FCL-PEG NSs as PA agents. As illustrated in Fig. 3d, FCL-PEG NSs demonstrated high photothermal conversion efficiency in vitro. The feasibility of FCL-PEG NSs as photothermal imaging agents was also assessed in vivo. After 24 h i.v. injection of FCL-PEG NSs, the tumor sites of mice were irradiated with 658 and 808 nm lasers for 10 min. Temperature increases at tumor sites were recorded using an IR thermal camera (Fig. 7c and d). Compared with the control group and lasers-only groups, a slight temperature increase (5.9 °C) and a higher temperature increase (21.0 °C) at tumor sites were observed under treatment of 658 nm (0.5 W cm^−2^) and 808 nm (1.0 W cm^−2^) laser irradiation, respectively, after 24-h injection of FCL-PEG NSs. The prominent temperature increases at tumor sites reflect the huge potential of FCL-PEG NSs as photothermal imaging agents in vivo.

Based on their cytotoxicity to specific cancer cells and multi-modal antitumor performance in vitro, FCL-PEG NSs-based combinatorial cancer therapies were assessed in vivo (Fig. 7a). HepG2 tumor-bearing mice were treated as follows. Group 1: PBS solution; Group 2: 808 nm + 658 nm laser irradiations; Group 3: FCL-PEG NSs; Group 4: FCL-PEG NSs + 658 nm laser; Group 5: FCL-PEG NSs + 808 nm laser; and Group 6: FCL-PEG NSs + 658 nm + 808 nm. The power intensity of 658 and 808 nm lasers were 0.5 and 1.0 W cm^−2^, respectively. Irradiation time was 10 min after 24 h i.v. injection of FCL-PEG NSs (5 mg/kg). Figure 7e presents tumor volume growth under different treatments. Fast tumor growth was observed in the control group and lasers-only group, meaning laser irradiation alone (658 and 808 nm) had almost no effect on tumor growth. For the FCL-PEG NSs treated group, there was slight inhibition of tumor growth, further confirming specific cancer cell cytotoxicity through ·OH generation via Fenton reaction of Fe_2_O_3_. For treatments combining FCL-PEG NSs with 658 or 808 nm lasers, more distinct inhibition of tumor growth was observed, demonstrating the strong antitumor effect of PTT and photo-enhanced CDT/PDT of FCL-PEG NSs with corresponding laser irradiation. As expected, the tumors were completely ablated in Group 6, which further demonstrated the potential of FCL-PEG NSs as antitumor photomedicines. After 14 days of treatments, tumors were excised and examined (Fig. 7f), providing direct evidence for the prominent therapeutic effect in the photo-enhanced CDT/PDT/PTT group. Moreover, there was no obvious effect on the mice’s weight in these six treatments (Fig. 7g).

### Biocompatibility evaluation of FCL-PEG NSs

**Fig. 8:**
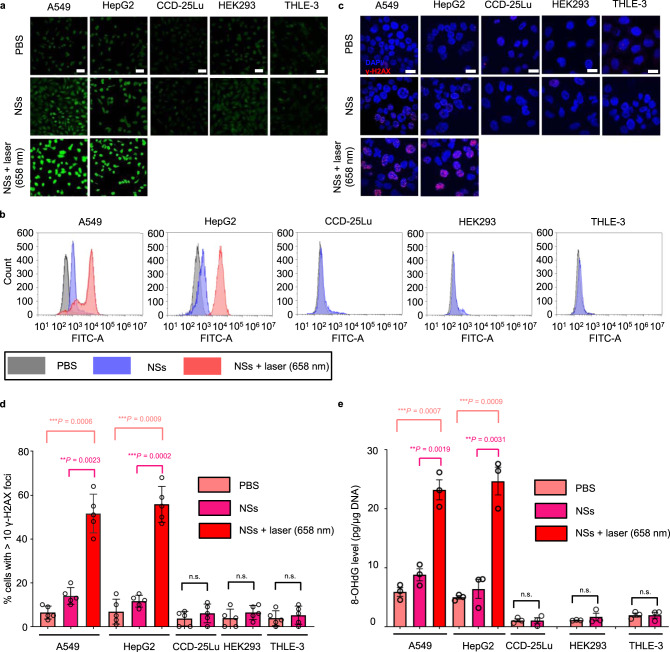
In vitro toxicity. **a** Representative confocal microscopy images (three times each experiment was repeated independently with similar results) and **b** flow cytometry detection of ROS contents from the different cancer cell lines and normal cell lines (scale bars, 50 μm) after different treatments (irradiation was performed only on cancer cells). **c** Representative confocal microscopy images of the different cancer cell lines and normal cell lines (scale bars, 50 μm) after different treatments. The nuclei were stained by DAPI (blue), and the γH2AX foci per nucleus were stained by anti-γH2AX antibody (red). Five times each experiment was repeated independently with similar results. **d** Quantification of the percentages of cells with >10 γH2AX foci numbers by confocal microscopy. The data show mean ± s.d., *n* = 5 biologically independent cells, and significance was determined using a two-tailed *t*-test (**P < 0.01, ***P < 0.001). **e** In vitro DNA damage of the cells after different treatments were measured by 8-OHdG assay. The data show mean ± s.d., *n* = 3 biologically independent cells, and significance was determined using a two-tailed *t*-test (**P < 0.01, ***P < 0.001).

Although the main ingredients of FCL NSs (MgO and Fe_2_O_3_) were FDA-approved therapeutics, in this section, we describe further confirmation of the biocompatibility of FCL-PEG NSs both in vitro and in vivo through a series of detailed analyses. It is worth mention that previous studies have indicated that oxygen vacancy on the surface of the FCL-PEG NSs might generate ·O_2_^−^ from O_2_ and lead to potential toxicity for normal tissues. However, specific cytotoxicity to cancer cells and good biocompatibility with normal cells were observed (Fig. 5a and b). The reason for the good biocompatibility may be the limited oxygen vacancy on the surface of FCL-PEG NSs and short lifetime of ·O_2_^−^. Moreover, the main reason for the specific cytotoxicity of FCL-PEG NSs to cancer cells could be the high H_2_O_2_ content in TME, which facilitated the Fenton reaction of FCL-PEG NSs and generated more cytotoxic ·OH. To test this possibility, we further analyzed the effect of FCL-PEG NSs on ROS production in both cancer cells and normal cells. The cancer cells (i.e., A549 and HepG2) and normal cells (i.e., CCD-25Lu, HEK 293, and THLE-3) were treated with FCL-PEG NSs for 24 h and cultured with 2′,7′-dichlorodihydrofluorescein diacetate (DCFH-DA) before measurement of ROS levels using CLSM and flow cytometry (FCM). As shown in Fig. 8a and b, the FCL-PEG NSs obviously increased intracellular ROS levels in two cancer cell lines compared to normal cell lines. The specific ROS generation effect in cancer cells was distinct and detectable following 24-h incubation using FCL-PEG NSs both via CLSM and the FCM. Besides, much higher ROS levels were observed in cancer cell lines after 658 nm laser irradiation for 10 min. This phenomenon further demonstrated that FCL-PEG NSs with suitable energy band structures could act as effective photosensitizers for PDT and targeted tumor therapy. More importantly, the toxicity (or therapeutic effect) occurred only in the cancer cells but not normal cells through both internal stimulus (i.e., high content of H_2_O_2_ in TME) and external stimulus (658 nm laser irradiation; only performed within cancer sites)^53^, guaranteeing the safety of this therapeutic strategy and minimizing side effects (i.e., biocompatible in therapeutic applications). As previously reported^54^, the reason for ROS-induced cell toxicity is mainly DNA damage caused by ROS. We thus further analyzed the levels of DNA damage in different cell lines after treatment with FCL-PEG NSs using γ-H2AX as a marker for DNA double-strand breaks^55^. As shown in Fig. 8c and d, normal cell lines treated with FCL-PEG NSs alone did not show any obvious DNA damage, while cancer cell lines showed detectable levels of DNA damage, further supporting the biocompatibility of FCL-PEG NSs in normal cells and specific toxicity in cancer cells. Treatment with FCL-PEG NSs and 658 nm laser irradiation-induced remarkably high levels of irreparable DNA damage in the cancer cells. To further demonstrate this potential mechanism, 8-hydroxy-2′-deoxyguanosine (8-OHdG), a reliable marker for oxidative stress^56^, was detected in both cancer cell lines and normal cell lines after treatment with FCL-PEG NSs. As displayed in Fig. 8e, the 8-OHdG results showed trends consistent with the γ-H2AX data. These results together further confirmed that the therapeutic strategy based on FCL-PEG NSs can specifically kill cancer cells, while maintaining good biocompatibility with normal cells.

**Fig. 9:**
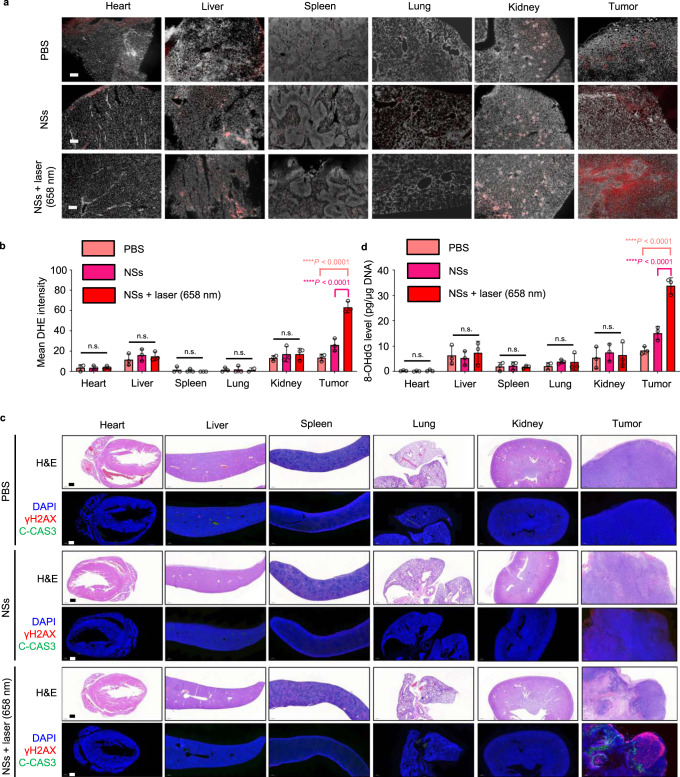
In vivo toxicity. **a** In vivo ROS detection in the sections from major organs and tumors by dihydroethidium (DHE) via fluorescence microscopy. The major organs and tumors were collected from the HepG2 xenograft-tumor-bearing mice after different treatments with PBS, NSs, or NSs + 658 nm NIR irradiation (irradiation was performed only within tumor areas). Scar bar = 200 µm. **b** Quantification of the in vivo ROS signals from the major organs and tumors calculated from the section studies in (**a**). The data show mean ± s.d., *n* = 3 biologically independent mice, and significance was determined using a two-tailed *t*-test (****P < 0.0001). **c** H&E staining and immunofluorescence (IF) staining in the sections from the major organs and tumors, which were collected from the HepG2 xenograft-tumor-bearing mice after different treatments with PBS, NSs, or NSs + 658 nm NIR irradiation (the irradiations were only performed within tumor areas). The nucleus was stained by DAPI (blue), damaged DNA was stained by γH2AX foci (red), and apoptotic cells were stained by apoptosis marker C-CAS3 (green). Scar bar = 500 µm. Three times each experiment was repeated independently with similar results. **d** In vivo DNA damage of the major organs and tumors after different treatments was measured by 8-OHdG assay. The data show mean ± s.d., *n* = 3 biologically independent mice, and significance was determined using a two-tailed *t*-test (****P < 0.0001).

To further validate selective toxicity to tumors and good biocompatibility with normal tissues of the FCL-PEG NSs-based therapeutic strategy in vivo, we further performed more detailed studies in mice bearing HepG2 xenograft tumors. First, to more precisely characterize the biodistribution of FCL-PEG NSs in vivo, an inductively coupled plasma emission spectrometer (ICP) was employed to analyze the concentration of FCL-PEG NSs in major organs and tumors over 30 days. Besides the accumulations in tumors, high accumulations of FCL-PEG NSs were also found in other normal organs such as liver, lung, and kidneys. However, the accumulated FCL-PEG NSs in normal organs could be gradually excreted by the body over time (Supplementary Fig. 19). Moreover, no obvious difference in ROS levels was observed in normal tissues after treatment with FCL-PEG NSs compared with those treated with PBS (Fig. 9a and b), indicating likely biocompatibility of the FCL-PEG NSs in normal tissues (i.e., the limited oxygen vacancy on the surface of FCL-PEG NSs cannot cause obvious toxicity in normal tissues). We also observed higher levels of ROS in tumor sections in the FCL-PEG NSs group compared with those of the PBS group, further confirming that the high H_2_O_2_ content in TME could facilitate the Fenton reaction of FCL-PEG NSs and generate more cytotoxic ·OH in tumor sites. Treatment with FCL-PEG NSs and 658 nm laser irradiation within the tumor sites significantly and specifically induced generation of ROS levels only in tumors, further indicating the safety of this therapeutic strategy with minimal side effects. Next, we assessed DNA damage levels through γ-H2AX staining, apoptosis levels through cleaved caspase-3 (C-CAS3) staining, and performed histological analyses through H&E staining in vivo. As shown in Fig. 9c and d, no detectable DNA damage, no detectable apoptosis, and no detectable signs of organ injury were identified in normal tissues, confirming in vivo biocompatibility of the FCL-PEG NSs. Higher levels of DNA damage and apoptosis in tumor sections were observed after treatment with FCL-PEG NSs, especially when combined with 658 nm laser irradiation. Hence, these results clearly demonstrated that this therapeutic strategy based on FCL-PEG NSs can specifically target the tumor tissues in vivo while maintaining biocompatibility with normal tissues. Moreover, hematology assay, histology examination, and immune analysis were also employed to further demonstrate the biocompatibility of FCL-PEG NSs. For the hematology and histology assay, routine blood examination was conducted 1, 7, and 14 days after i.v. injection of FCL-PEG NSs (10 mg/kg). As shown in Supplementary Fig. 20a–f, the amount of creatinine, aspartate aminotransferase (AST), alanine aminotransferase (ALT), albumin, total protein (TP), and blood urea nitrogen (BUN) were not statistically different from those in the control group. For immune toxicity analysis, the amount of IFN-γ, IL-6, and TNF-α in serum samples from mice after 12 and 24 h post i.v. injection of FCL-PEG NSs (10 mg/kg) were measure. Supplementary Fig. 20g–i shows that all cytokine levels were nearly the same as those in the control group. These data further confirmed that the obtained FCL-PEG NSs are biocompatible in vivo.

### Storage stability evaluation of FCL-PEG NSs

Good stability of FCL-PEG NSs in the physical environment (e.g., in PBS or saline) will be a great advantage for the production, storage, and transportation of NSs-based therapeutics for medical use in the future. Therefore, in the final part of this study, we further investigated the storage stability of FCL-PEG NSs under normal physiological conditions. The FCL-PEG NSs were dispersed in PBS at 25 °C for 30 days, and then their microscopic morphology and optical property changes were assessed at varied time intervals (0, 1, 2, 3, 4, 5, 6, 7, 10, and 30 days). As shown in Supplementary Fig. 21a and b, there was a negligible change in the corresponding vis–NIR absorption spectra and morphology of the FCL-PEG NSs suspension, indicating excellent storage stability. As Fe_2_O_3_ and MgO were the principal components of FCL-PEG NSs, we went on to check whether TME (such as low pH and high H_2_O_2_)-responsive degradation could be observed. The FCL-PEG NSs were dispersed in PBS with pH 5.5 or 50 μM H_2_O_2_, and their microscopic morphology and any changes in optical properties were assessed at varied time intervals (0, 1, 2, 3, 4, 5, 6, 7, 10, and 30 days). A significantly decreasing absorbance intensity with increasing duration of dispersion was observed in their corresponding vis–NIR absorption spectra (Supplementary Fig. 21c and e), in which nearly 50% and 80% of FCL-PEG NSs were degraded in 50 μM H_2_O_2_ and pH 5.5 PBS, respectively. Moreover, TEM images exhibited that the microstructure of FCL-PEG NSs was damaged to varying degrees (Supplementary Fig. 21d and f). Therefore, besides their high storage stability, TME-triggered biodegradation of FCL-PEG NSs was also observed. Considering that (i) injected FCL-PEG NSs in major organs can be excreted from the body gradually (Supplementary Fig. 19) and those in tumors can be degraded (i.e., biocompatibility), (ii) the main ingredients of FCL-PEG NSs are FDA-approved (i.e., biocompatibility and clinical potential), and (iii) the excellent storage stability of the FCL-PEG NSs (i.e., an advantage in the production, storage, and transportation), the developed FCL-PEG NSs may possess great potential in biomedical applications.

## Discussion

In recent years, several nanomedicines have been developed and demonstrated with great therapeutic effects in various cancers: 1D Fe_2_P nanorods acting as Fenton agents in response to NIR II Light and ultrasound for deep tumor synergetic theranostics^57^, 2D black phosphorus NSs used as photothermal and photodynamic agents in response to vis/NIR I Light for photonic therapies^58–60^, and 3D FeS_2_ nanoparticles serving as photonic and Fenton agents in response to vis/NIR I light for photothermal-enhanced CDT/PDT^51,61,62^. Compared with other nanomedicines, FCL-PEG NSs captured from sandwich-structured VMT via selectively alkali-etching the “bread layer” may have the following advantages. For example, the main components of FCL-PEG NSs (MgO and Fe_2_O_3_) are FDA-approved. The raw materials are cheap and widely sourced, and the efficient preparation method has high universality, which is appropriate for all-natural and synthetic 2:1 aluminosilicates. Moreover, the NSs alone integrate the regulation of TME, PTT/PDT/CDT, and multi-mode imaging, serving as an efficient and comprehensive nanomedicine for cancer theranostics.

It is worth to mention that, the FCL-based NSs developed in this study also present a good example exhibiting the evolution from a commonly used traditional Chinese medicine (i.e., the raw material VMT) to a new photonic nanomedicine, which is enabled by nanotechnology and materials science-based technology. Future studies in the development of traditional Chinese medicine may be greatly inspired by such kind of perspective from the emerging nanotechnology and materials science fields^63^. More interestingly, without the sandwiched structures that shielded active sites, the obtained NSs from VMT exhibit a tunable and appropriate electron band structure with the bandgap decreased from 2.0 to 1.4 eV, a conductive band increased from −0.4 to −0.6 eV, and excellent light response characteristics. The great properties of 2D FCL NSs may endow them with exciting potential in diverse applications such as energy, photo/electro-catalysis, and biomedical engineering.

In summary, a smart strategy was developed to produce 2D ultrathin FCL NSs (effective constituent: Fe_2_O_3_ and MgO) from sandwich-structured hydro-aluminosilicate via selectively degrading the “bread layer” (effective constituent: SiO_2_ and Al_2_O_3_). The prepared FCL NSs with an average thickness and size of 2.7 and 110 nm, respectively, possessed a tunable and appropriate electron band structure with the bandgap decreased from 2.0 to 1.4 eV and the conductive band increased from −0.4 to −0.6 eV. The captured 2D FCL NSs exhibited a high photothermal conversion efficiency serving as promising photothermal agents. Benefiting from the narrowed bandgap and improved ΔE between the CB of FCL-PEG NSs and E^0^ of O_2_/·O_2_^−^, effective electron-hole separation of FCL NSs was obtained upon 658 nm laser irradiation, which facilitated ·O_2_^−^ generation and PDT efficacy. Moreover, the capacity of FCL-PEG NSs for the generation of ·OH in TME via Fenton reaction could be significantly enhanced by 808 and 658 nm laser exposures, which further endowed FCL-PEG NSs with potential for photo-enhanced CDT. In addition, FCL-PEG NSs had a strong ability to modulate TME through catalyzing H_2_O_2_ to produce O_2_ and consuming GSH, which could relieve hypoxia and diminish the antioxidant capability of the tumor. FCL-PEG NSs also showed great utility in PA, photothermal, and fluorescent imaging.

Considering that MgO and Fe_2_O_3_ therapeutics are already FDA-approved and have been widely used in the clinic for different diseases, the innovative 2D FCL layers developed in our study are biocompatible and potentially highly impactful in terms of basic science and translational medicine. This work is also expected to supply a smart strategy for the preparation of 2D nanomaterials from 2:1 aluminosilicate with tunable electron band structure and inspire future studies that expand their in-depth application, especially in biomedicine, energy, catalysis, and biomedical engineering.

## Methods

### Materials

Vermiculite was supplied from Yunnan province, China. Cy7-PEG-NH_2_ (MW: 5k) and PEG-NH_2_ (MW: 5k) were purchased from Nanocs Inc. PBS (pH 7.4 and 5.5), DMEM medium, RPMI medium, trypsin-EDTA, and fetal bovine serum (FBS) were purchased from Gibco Life Technologies. 9,10-anthracenedipropanoic acid (ABDA), [Ru(dpp)_3_]Cl_2_ (RDPP), H_2_O_2_ (30%), N-methyl-pyrrolidone (NMP), methylene blue (MB), 3,3′,5,5′-tetramethylbenzidine (TMB), glutathione, 5,5′-dithiobis (2-nitrobenzoic acid) (DTNB), and dihydrorhodamine 123 (DHR123) were supplied by Sigma-Aldrich.

### Synthesis of FCL NSs from VMT

FCL NSs were prepared by coupling ball-milling, calcination, NaOH etching, and probe sonication. First, VMT powders (500 mg) were dispersed into an NMP solution (100 mL), and then the solution was ground for 30 min. After washing and drying, the ground VMT powders were calcined at 800 °C for 2 h in a furnace under N_2_ atmosphere. Next, the calcined VMT powders were immersed in a saturated NaOH solution (100 mL) in a sealed autoclave. The sealed autoclave was then placed in a furnace preheated to 150 °C for 24 h. The NaOH etched VMT solution was then centrifuged and washed three times to remove any residual NaOH, and then ultrasonicated for 5 h in the NMP solution. After sonication, the VMT solution was centrifuged at 1006 × *g* to remove the unexfoliated VMT. The supernatant was centrifuged at 16,099 × *g* and washed three times with DI water.

### Surface coating of FCL NSs

PEG-NH_2_ (10 mg) was dissolved into the FCL NSs solution (10 mL, 0.1 mg/mL) and stirred for 12 h after 30 min of sonication. Then the solution was centrifuged for 30 min at 698 × *g* (4 °C) with Amicon pipes (MWCO 100 kDa; Millipore) and the precipitates were washed three times to remove the residual PEG-NH_2_. The same process was used for fluorescent modification of FCL-PEG-Cy7 NSs using Cy7-PEG-NH_2_ in a dark environment.

### Characterization

Transmission electron microscopy (TEM, JEM-2100UHR, JEOL, Japan) and scanning electronic microscopy (SEM, JSM-6700F, JEOL, Japan) were used for direct observation of the morphology of NSs. Atomic-force microscopy (AFM, FASTSCANBIO, Germany) and dynamic light scattering were applied to characterize the thickness and size of the NSs. The chemical constituent of FCL NSs was detected via energy-dispersive X-ray spectroscopy (EDX) (Inca X-MAX, Oxford, UK), Fourier-transform infrared spectrophotometry (FTIR, Nexus 470, Nicolet, Madison, WI, USA), and X-ray photoelectron spectroscopy (XPS, ESCALAB 250Xi, Japan). The absorption of FCL NSs and VMT powder was detected by solid UV–vis-NIR spectrophotometer (Hitachi, UH4150, Japan).

### The calculation process of photothermal conversion efficiency

The photothermal conversion efficiency of FCL-PEG NSs was calculated by the following formula.

Based on the total energy balance for this system:

1 $$\mathop {\sum }\limits_i m_iC_{{\mathrm{p}},i}\frac{{dT}}{{dt}} = Q_{{\mathrm{VMT}}\,{\mathrm{NSs}}} + Q_{\mathrm{s}} - Q_{{\mathrm{loss}}}$$

*T* stands for the temperature of the solution. *C*_p_ stands for the heat capacity. m stands for the mass of the solvent.

*Q*_FCL NSs_ stands for the photothermal energy produced by FCL-PEG NSs:

2 $$Q_{{\mathrm{FCL}}\,{\mathrm{NSs}}} = I(1 - 10^{ - A_{808}})\eta$$

Laser power was expressed by *I*, the absorbance of FCL-PEG NSs under 808 nm laser irradiation is expressed by *A*_808,_ and the conversion efficiency is expressed by *η*.

The light absorption heat of water is expressed by *Q*_s_.

Thermal energy delivers to the environment is expressed by *Q*_loss_.

3 $$Q_{{\mathrm{loss}}} = hA\Delta T$$

The temperature change is expressed by Δ*T*, the surface area of the container is expressed by *A* and the heat transfer coefficient is expressed by *h*.

At the maximum steady-state temperature, the heat output is equal to the heat input, which is:

4 $$Q_{{\mathrm{FCL}}\,{\mathrm{NSs}}} + Q_{\mathrm{s}} = Q_{{\mathrm{loss}}} = hA\Delta T_{{\mathrm{max}}}$$

The temperature change at the maximum steady-state temperature is expressed by Δ*T*_max_. Based on formulas 2 and 4, the *η* can be calculated as follows:

5 $$\eta = \frac{{hA\Delta T_{{\mathrm{max}}} - Q_{\mathrm{s}}}}{{I(1 - 10^{ - A_{808}})}}$$

To the value of *hA*, *θ* is used to represent the ratio of Δ*T* to Δ*T*_max_:

6 $$\theta = \frac{{\Delta T}}{{\Delta T_{max}}}$$

Formula 7 was substituted into formula 2, and formula 2 was rearranged:

7 $$\frac{{d\theta }}{{dt}} = \frac{{hA}}{{\mathop {\sum }\nolimits_i m_iC_{{\mathrm{p}},i}}}\left[ {\frac{{Q_{{\mathrm{FCL}}\,{\mathrm{NSs}}} + Q_{\mathrm{s}}}}{{hA\Delta T_{{\mathrm{max}}}}} - \theta } \right]$$

Shutting off the laser, *Q*_NSs_ + *Q*_s_ = 0, so formula 8 turn into:

8 $$dt = - \frac{{\mathop {\sum }\nolimits_i m_iC_{{\mathrm{p}},i}}}{{hA}}\frac{{d\theta }}{\theta }$$

As shown below, formula 8 was integrated:

9 $$t = - \frac{{\mathop {\sum }\nolimits_i m_iC_{{\mathrm{p}},i}}}{{hA}}\theta$$

Thus, *hA* can be measured by experiment. *η* of FCL-PEG NSs can be obtained by substituted *hA* value into formula 6.

### Photothermal performance of FCL-PEG NSs

A series of concentrations (ranging from 0.05 to 0.2 mg/mL) of FCL-PEG NSs solution was exposed to an 808 nm laser at different power densities (ranging from 0.5 to 2 W/cm^2^) for 5 min. The increase in temperature of the FCL-PEG NSs solution was detected using an IR thermal camera.

### GSH degradation in vitro

First, DTNB (0.2 mg/mL) solution was mixed with GSH (final concentration: 0.1 mM) solution. Next, the FCL-PEG NSs, with final concentrations ranging from 0.025 to 0.2 mg/mL, were added into the above solution and reacted for 30 min. UV–vis spectroscopy was applied to record the absorbance of the DTNB every 5 min.

### Extracellular O_2_ Production

FCL-PEG NSs at a final concentration of 0.2 mg/mL were added into the H_2_O_2_ solution with the final concentration of 0.1 mM. The O_2_ generated from this solution was measured by a dissolved oxygen meter.

### ·OH production in vitro

First, FCL-PEG NSs and MB were fully mixed in PBS (pH 7.4) and stirred for 1 h in a dark environment. After that, H_2_O_2_ (150 μL, 10 mM) was added to the suspension, for final concentrations of FCL-PEG NSs and MB 0.025 and 0.05 mg/mL, respectively. For photo-enhanced CDT detection, the solution was exposed to 808 and 658 nm lasers (1 and 0.5 W/cm^2^) irradiation. Degradation of MB was determined by detecting the absorbance of the supernatant via UV–vis spectroscopy.

### ·O_2_^−^ production in vitro

First, FCL-PEG NSs (0.1 mg/mL) and DHR123 (1 μL, 1 mM) were fully mixed in PBS (pH 7.4) and stirred for 1 h in a dark environment. Then the reaction solution was exposed to a 658 nm laser (0.5 W/cm^2^). The generated fluorescence of DHR123 was detected by fluorescence spectrophotometer.

### Electron paramagnetic resonance (EPR) for detecting ·OH and ·O_2_^−^ in vitro

To further detect the generation of ·OH and ·O_2_^−^, FCL-PEG NSs (0.2 mg/mL), and 5,5-dimethyl-1-pyrroline N-oxide (DMPO) (0.1 mM) were fully mixed (solution for testing ·OH: DI water, solution for testing ·O_2_^−^: methanol). The ·OH and ·O_2_^−^ signals were recorded by EPR.

### Cytotoxicity of FCL-PEG NSs

Both normal and cancer cells were plated and cultured in 96-well plates (37 °C, 5% CO_2_) for 24 h. Then the FCL-PEG NSs at different concentrations (ranging from 0.025 to 0.2 mg/mL) were added to the culture medium and the cells were further cultured for another 24 h. Finally, cell viabilities were determined by MTT assay (Life Technologies) according to the manufacturer’s instructions.

### Degradation of GSH in cells

HepG2 cells were plated in 96-well plates and cultured for 24 h (37 °C, 5% CO_2_). Then the FCL-PEG NSs at different concentrations (ranging from 0.025 to 0.2 mg/mL) were added to the culture medium and the cells were further cultured for another 24 h. Then, the cells were washed with PBS and centrifuged to gather the precipitate. Subsequently, the cells were broken by an ultrasound cell crusher and the content of GSH in the supernatant detected using a GSH assay kit.

### Production of O_2_ in cells

HepG2 cells were planted on the culture dishes and cultured for 24 h (37 °C, 5% CO_2_). Then, the FCL-PEG NSs (final concentration of 0.2 mg/mL) and RDPP (final concentration of 1 μM) were added into the culture medium and further cultured for another 24 h. Finally, the concentrations of O_2_ in cells were detected by CLSM, after removing the culture medium and washing with PBS.

### Production of ROS in cells

HepG2 cells were planted on the culture dishes and cultured for 24 h (37 °C, 5% CO_2_). Then the FCL-PEG NSs (final concentration of 0.2 mg/mL) were added into the culture medium and cultured for another 24 h. Next, the DCFH-DA solution (final concentration of 0.2 μM) was added into the above medium and cultured for 0.5 h. After removing the culture medium and washing with PBS, the cells were irradiated for 10 min with a 658 nm laser with power 0.5 W/cm^2^. The green fluorescence induced by ROS was detected by CLSM.

### In vitro photo-enhanced chemodynamic, photodynamic, and photothermal therapy

HepG2 and A549 cells were plated in 96-well plates and cultured for 24 h (37 °C, 5% CO_2_). Then the FCL-PEG NSs at different concentrations (ranging from 0 to 0.2 mg/mL) were added into the culture medium and cultured for another 24 h. After removing the culture medium and washing with PBS, the cells were irradiated for 10 min by a 658 nm laser with power 0.5 W/cm^2^ or an 808 nm laser with a power of 1.0 W/cm^2^ for 10 min. After culturing the cells for 24 h, the cells were flushed by PBS three times. MTT assay was applied to detect cell viability.

### Pharmacokinetic study

For in vivo pharmacokinetic study, 200 μL of FCL-PEG-Cy7 NSs (6 mg/kg) were i.v. injected in C57BL/6 mice. After different intervals, 20 μL blood was gathered to test the fluorescence of Cy7 in blood via a microplate reader.

### In vivo imaging and biodistribution study

For in vivo fluorescence imaging and biodistribution study, 200 μL of FCL-PEG- Cy7 NSs (6 mg/kg) were i.v. injected into mice bearing HepG2 tumors. The Maestro2 In-Vivo Imaging System was used to detect the fluorescence of tumor and major organs 24 h post-injection. The fluorescence degree of Cy7 was measured via Image-J to quantify the concentration of NSs in major organs and tumors.

For in vivo PA imaging and biodistribution study, 200 μL of FCL-PEG NSs (6 mg/kg) were i.v. injected into mice bearing HepG2 tumors. The PA signal before injection was used as the control, and for imaging, the region of interest was 20 mm. All of the PA imaging was obtained using the Vevo LAZR photoacoustic imaging system (Visual-Sonics Co.).

### In vivo photo-enhanced chemodynamic, photodynamic, and photothermal therapy

After the tumors had grown to ~100 mm^3^, mice were randomly divided into 6 groups, 5 mice in each group. G1: saline as the control group, G2: 808 and 658 nm laser irradiation, G3: FCL-PEG NSs (6 mg/kg) (CDT), G4: FCL-PEG NSs with 658 nm laser (PDT combined with photo-enhanced CDT), G5: FCL-PEG NSs with 808 nm laser (PTT), and G6: FCL-PEG NSs with 658 and 808 nm laser irradiation (PDT combined with photo-enhanced CDT and PTT). The power density of 658 and 808 nm laser treatment was 0.5 and 1.0 W/cm^2^, respectively. The volume of tumors was recorded every 2 days during therapy, lasting 14 days.

### In vivo toxicity

C57BL/6 mice were i.v. injected with FCL-PEG NSs (10 mg/kg) for in vivo toxicity study. Main organs were collected and subjected to eosin (H&E) and hematoxylin staining at 30 days post-injection. C57BL/6 mice were i.v. injected with FCL-PEG NSs (10 mg/kg) to analyze the immune response. At 12 and 24 h post-injection, ELISA was applied to measure the concentrations of interleukin6 (IL-6), tumor necrosis factor-α (TNF-α), and interferon-γ (IFN-γ). At day 1, 7, and 14 after intravenous injection, urea nitrogen (BUN), creatinine (Cr), albumin (ALB), total protein (TP), aspartate alanine aminotransferase (ALT), and aminotransferase (AST) were measured in a complete blood panel.

### Statistical analysis

Graph Pad Prism 8.0 and Origin 9.0 were used for data statistics and statistical significance calculation. Microsoft Excel 2016 was used for biodistribution and tumor size analysis. Statistical analysis was performed using the Student’s *t*-test with statistical significance assigned at *P < 0.05 (significant), **P < 0.01 (moderately significant), ***P < 0.001 (highly significant), and ****P < 0.0001 (extremely significant).

### Reporting summary

Further information on research design is available in the Nature Research Reporting Summary linked to this article.

## Supplementary information

Supplementary Information

Reporting Summary

## Data Availability

The authors declare that all data supporting the findings of this study are available within the article and the Supplementary Information. Other data are available from the corresponding authors upon reasonable request.
