# Supplementary material for: Capturing functional two-dimensional nanosheets from sandwich-structure vermiculite for cancer theranostics
Source: Nat Commun. 2021 Feb 18;12:1124. doi: 10.1038/s41467-021-21436-5 (PMC7892577; doi:10.1038/s41467-021-21436-5)
Supplement: Supplementary file 1 — Supplementary Information [file 41467_2021_21436_MOESM1_ESM.pdf]

Supplementary Information

Capturing functional two-dimensional nanosheets from sandwich-structure vermiculite for cancer theranostics

Xiaoyuan Ji,[1,†] Lanlan Ge,[2,3,4] Chuang Liu,[1] Zhongmin Tang,[1] Yufen Xiao,[1] Wei Chen,[1] Zhouyue Lei,[5] Wei Gao,[5] Sara Blake,[1] Diba De,[1] Bingyang Shi,[6,7] Xiaobing Zeng,[2,*] Na Kong,[1,*] Xingcai Zhang,[5,*] Wei Tao[1,*]

[1] Center for Nanomedicine and Department of Anesthesiology, Brigham and Women's Hospital, Harvard Medical School, Boston, MA 02115, United States.

[2] Center Lab of Longhua Branch, Shenzhen People's Hospital, Second Clinical Medical College of Jinan University, Shenzhen, Guangdong 518120, China.

[3] Department of Infectious Disease, Shenzhen People's Hospital, Second Clinical Medical College of Jinan University, Shenzhen, Guangdong 518120, China.

[4] Integrated Chinese and Western Medicine Postdoctoral Research Station, Jinan University, Guangzhou, Guangdong 510632, China.

[5] School of Engineering and Applied Sciences, Harvard University, Cambridge, MA 02138, United States.

[6] Henan-Macquarie Uni Joint Centre for Biomedical Innovation, School of Life Sciences, Henan University, Kaifeng, Henan 475004, China.

[7] Department of Biomedical Sciences, Faculty of Medicine & Health Sciences, Macquarie University, Sydney, NSW 2109, Australia.

† Present address: Academy of Medical Engineering and Translational Medicine, Medical College, Tianjin University, Tianjin 300072, China.

Email: wtao@bwh.harvard.edu (W.T.); xingcai@mit.edu (X.Z.); nkong2@bwh.harvard.edu (N.K.); zeng.xiaobin@szhospital.com (X.Z.)

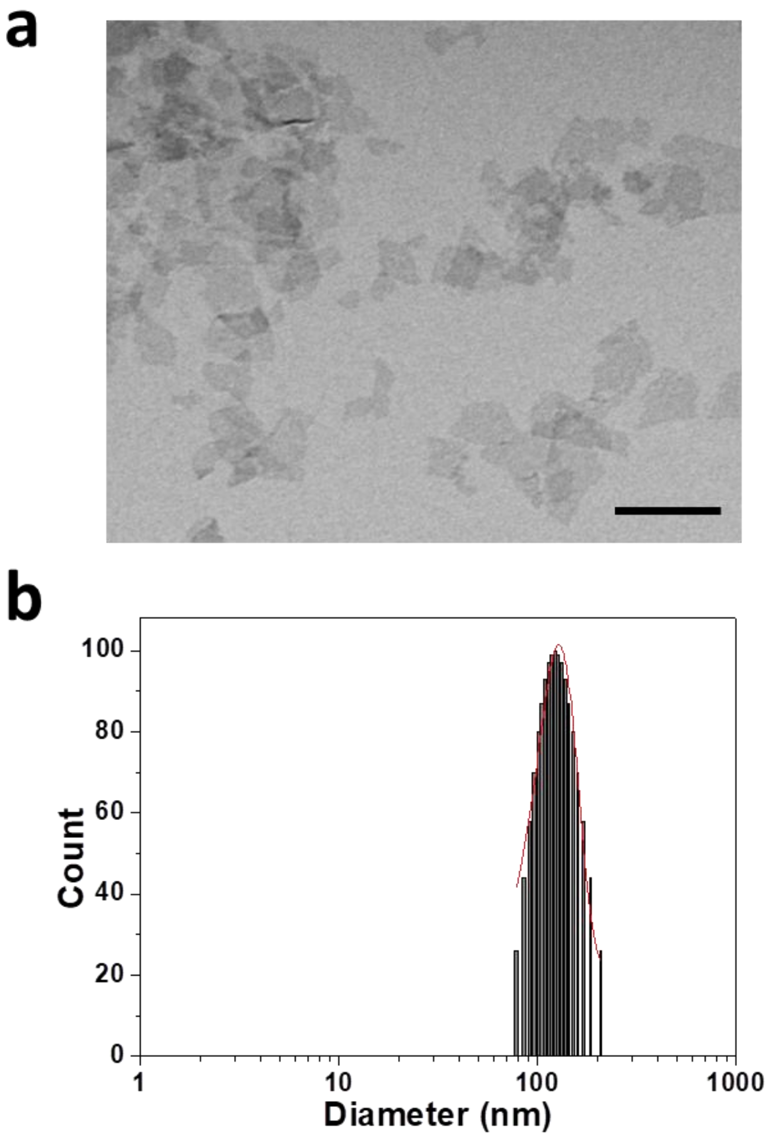

Supplementary Figure 1. Characterization of FCL NSs via TEM. **a** TEM images (three times each experiment was repeated independently with similar results), and **b** size distribution of the developed FCL NSs. The scale bar is 200 nm.

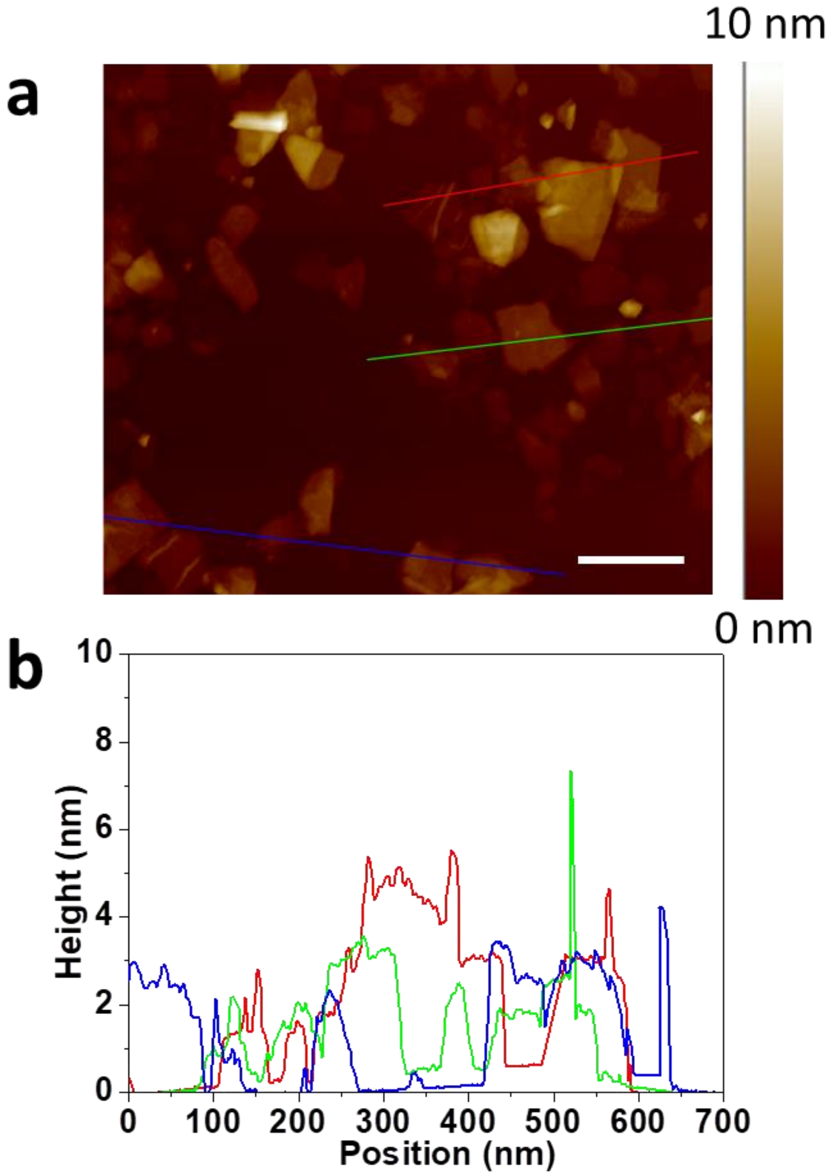

Supplementary Figure 2. Characterization of FCL NSs via AFM. **a** AFM images (three times each experiment was repeated independently with similar results), and **b** thickness of the developed FCL NSs. The scale bar is 200 nm.

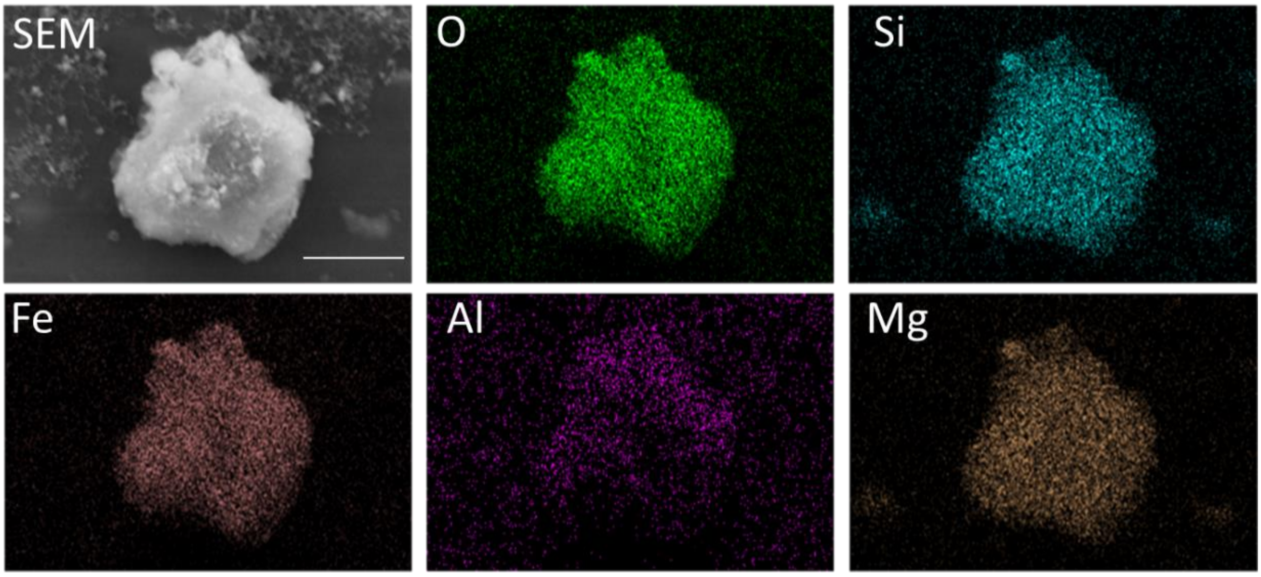

Supplementary Figure 3. STEM-EDS mapping images of VMT powder. Three times each experiment was repeated independently with similar results. The scale bar is 100 nm.

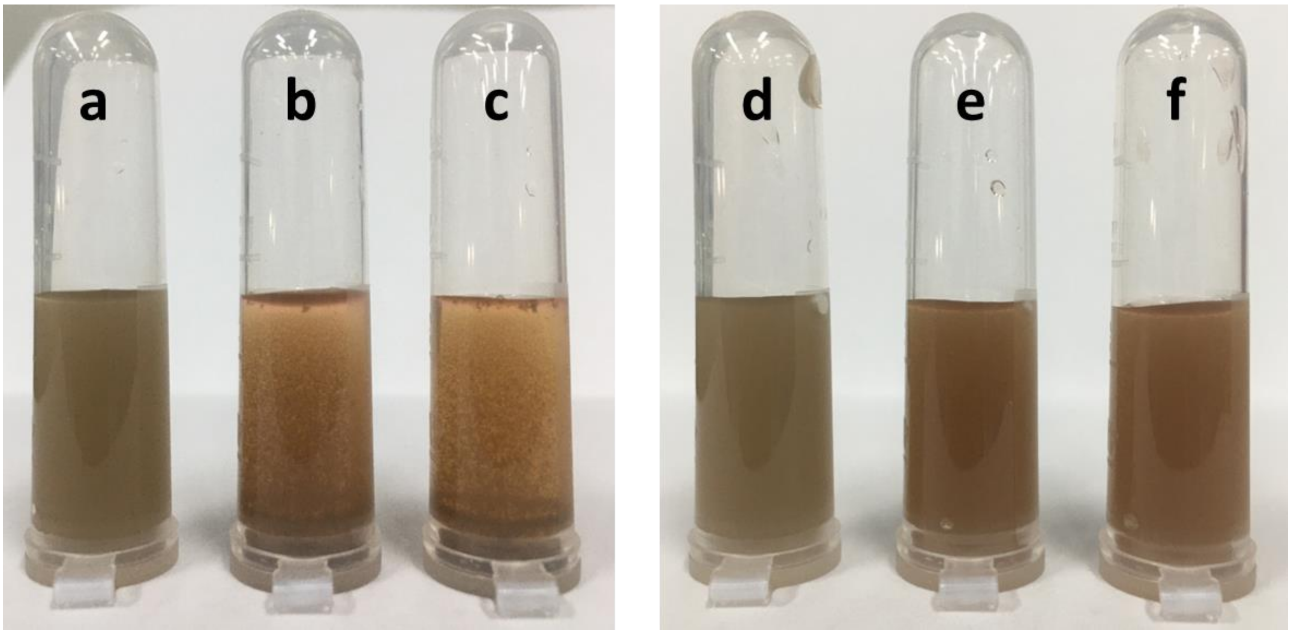

Supplementary Figure 4. The stability and dispersibility of FCL NSs and FCL-PEG NSs in different solutions. **a** FCL NSs in phosphate buffer saline (PBS). **b** FCL NSs in fetal bovine serum solution. **c** FCL NSs in Dulbecco's modified Eagle medium. **d** FCL-PEG NSs in PBS. **e** FCL-PEG NSs in fetal bovine serum solution. **f** FCL-PEG NSs in Dulbecco's modified Eagle medium.

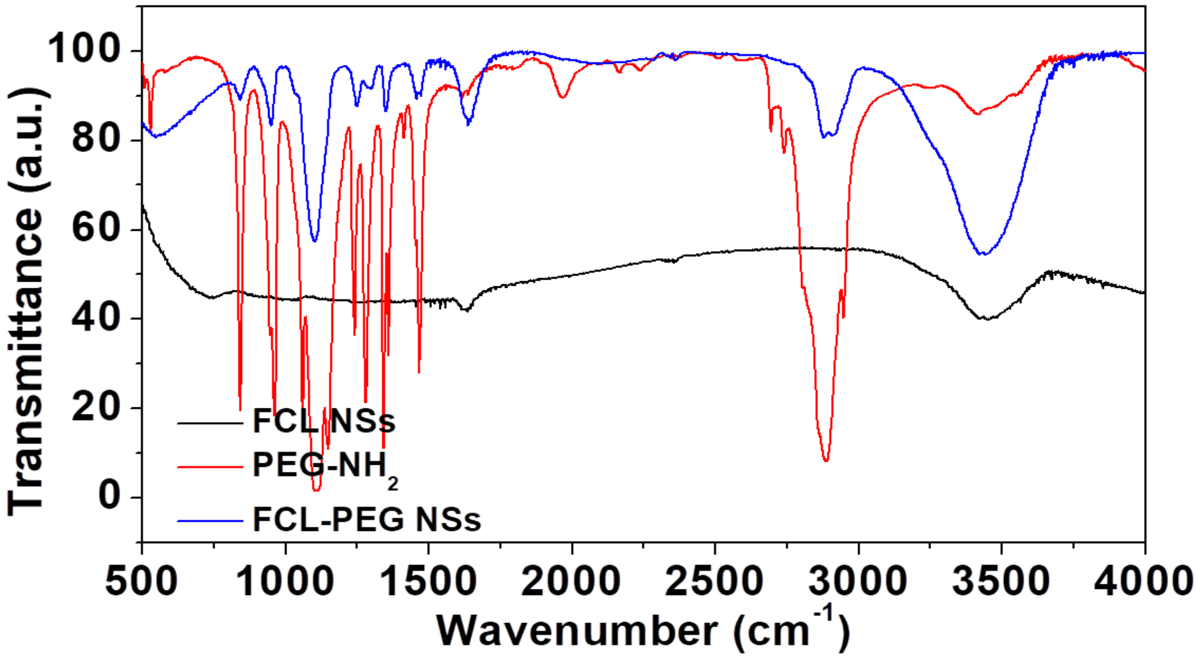

Supplementary Figure 5. FTIR spectrum of FCL NSs, PEG-NH$_2$, and FCL-PEG NSs.

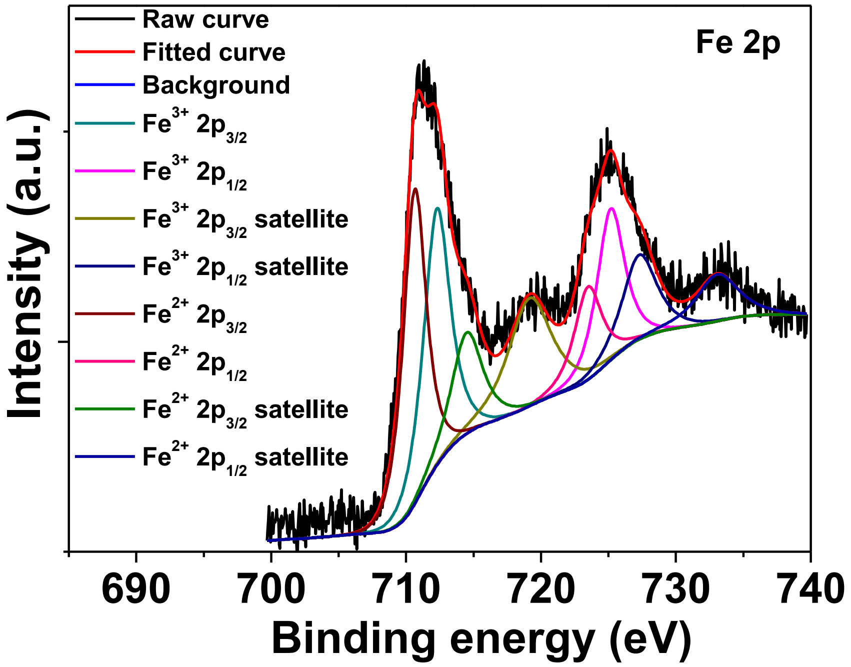

Supplementary Figure 6. HRXPS spectra of Fe 2*p* in FCL-PEG NSs.

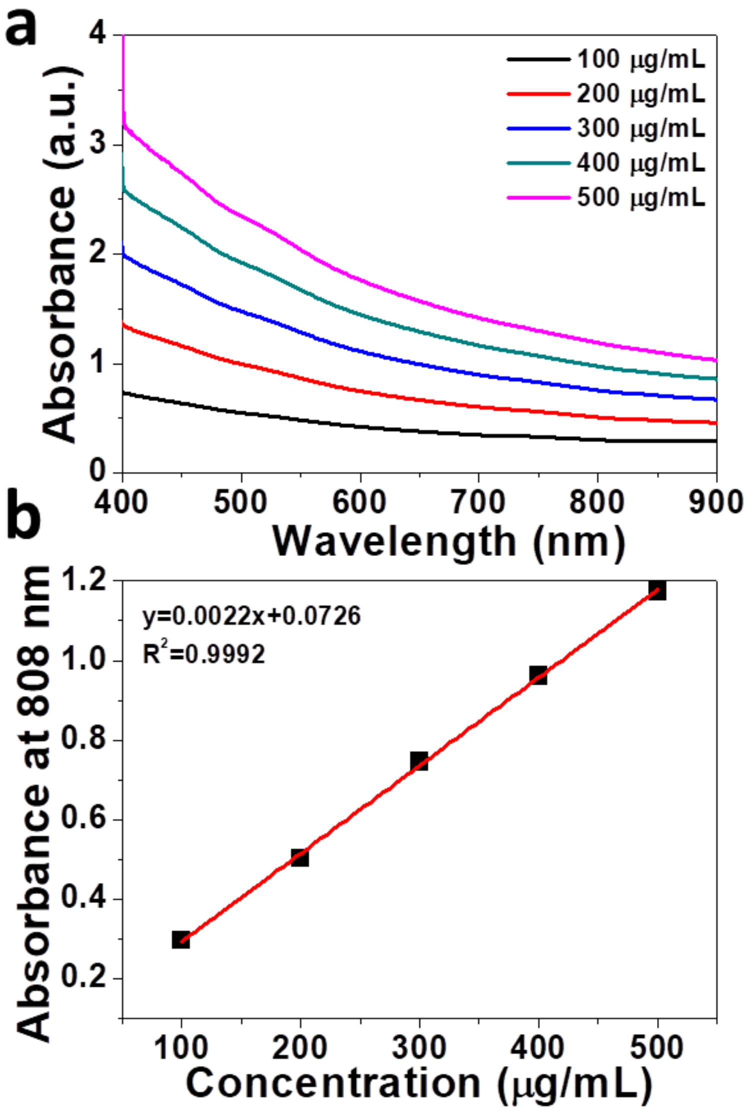

Supplementary Figure 7. Characterization of FCL NSs via absorbance. **a** The absorbance of FCL NSs. **b** Normalized absorbance intensity of FCL NSs at 808 nm.

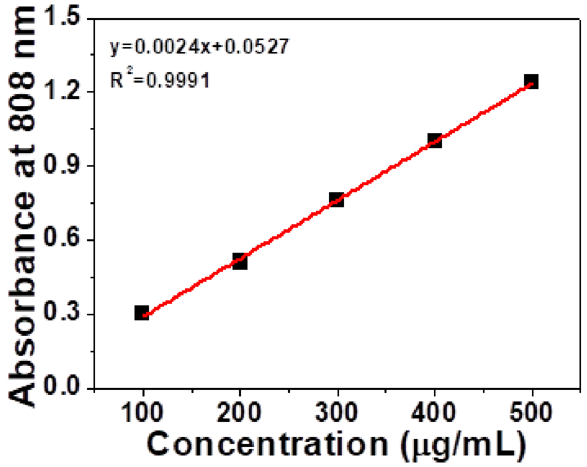

Supplementary Figure 8. Normalized absorbance intensity of FCL-PEG NSs at 808 nm.

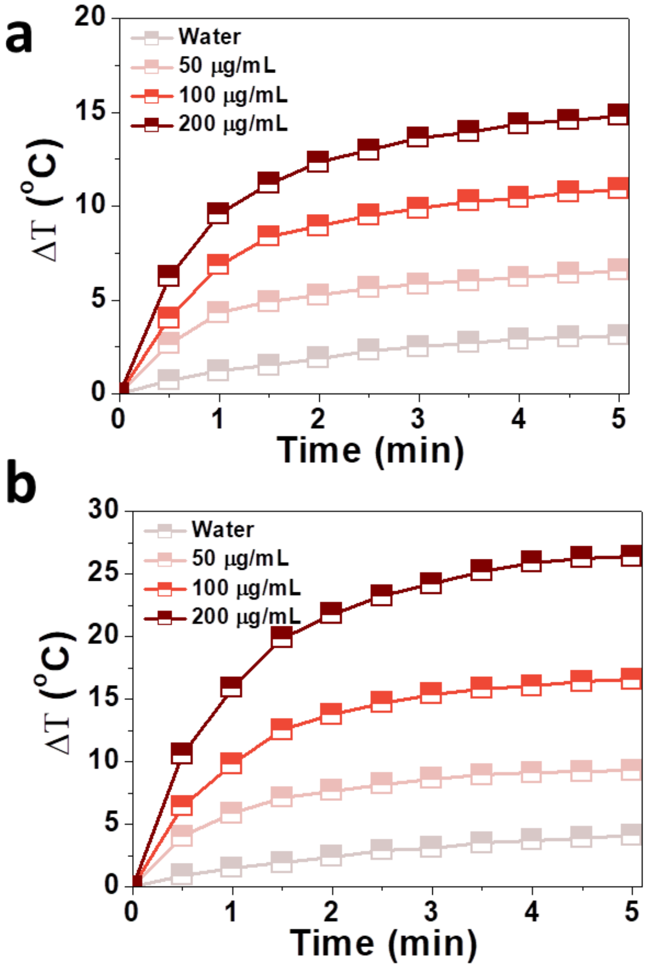

Supplementary Figure 9. Photothermal performance of FCL-PEG NSs. Temperature changes of different concentrations of FCL-PEG NSs solutions exposed to 808 nm laser at **a** 1 W/cm^2 and **b** 1.5 W/cm^2.

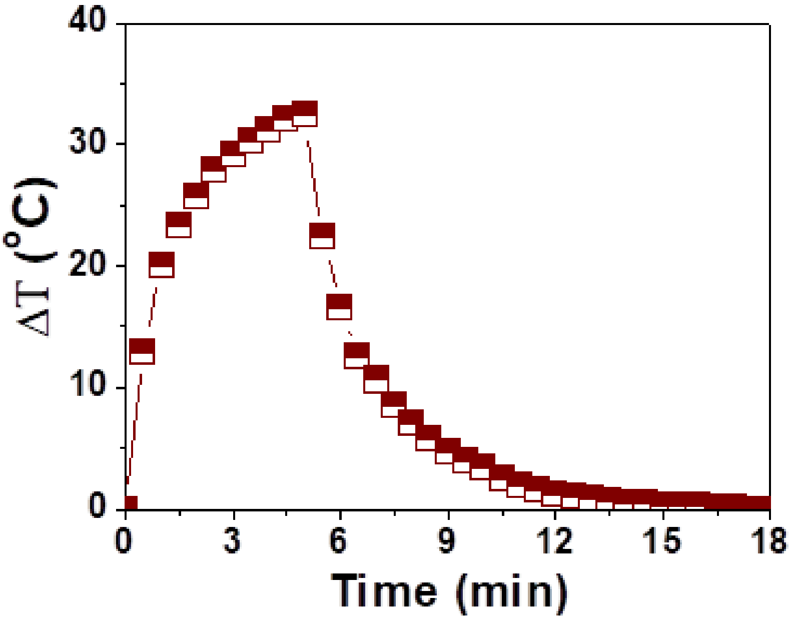

Supplementary Figure 10. The heating and cooling spectrum of FCL-PEG NSs solutions.

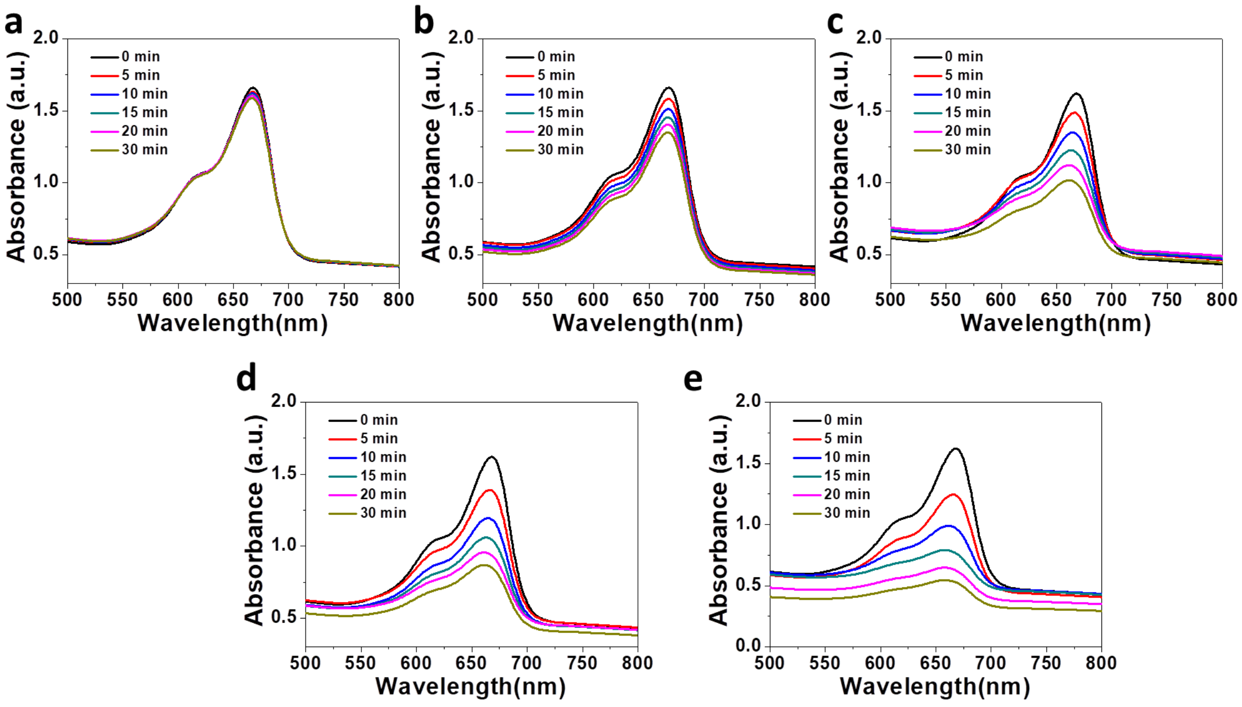

Supplementary Figure 11. Degradation of MB *via* generated ·OH. **a** 658 nm + 808 nm, **b** FCL-PEG NSs + H_2O_2, **c** FCL-PEG NSs + H_2O_2 + 808 nm, **d** FCL-PEG NSs + H_2O_2 + 658 nm, and **e** HOIL-PEG NSs + H_2O_2 + 658 nm + 808 nm. The concentrations of FCL-PEG NSs and H_2O_2 were 0.2 mg/mL and 250 μM, respectively. The power density of 658 nm and 808 nm lasers were 0.5 and 2 W/cm^2.

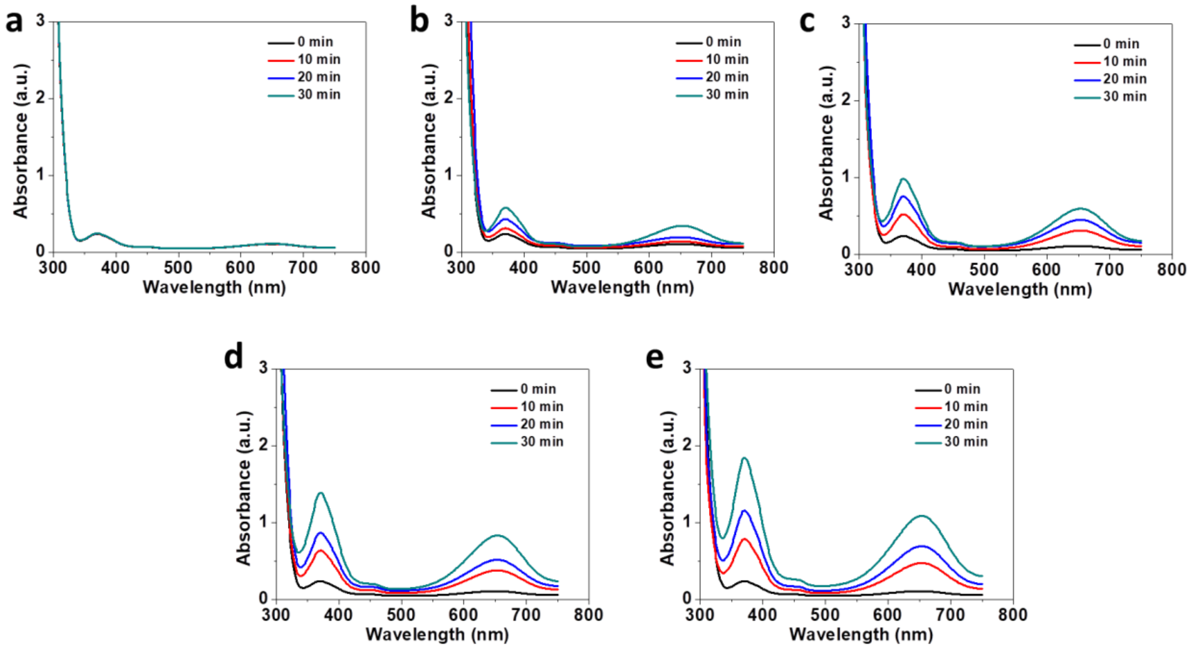

Supplementary Figure 12. Conversion of TMB *via* generated ·OH. **a** 658 nm + 808 nm, **b** FCL-PEG NSs + H_2O_2, **c** FCL-PEG NSs + H_2O_2 + 808 nm, **d** FCL-PEG NSs + H_2O_2 + 658 nm, and **e** HOIL-PEG NSs + H_2O_2 + 658 nm + 808 nm. The concentrations of FCL-PEG NSs and H_2O_2 were 0.2 mg/mL and 250 μM, respectively. The power density of 658 nm and 808 nm lasers were 0.5 and 2 W/cm^2.

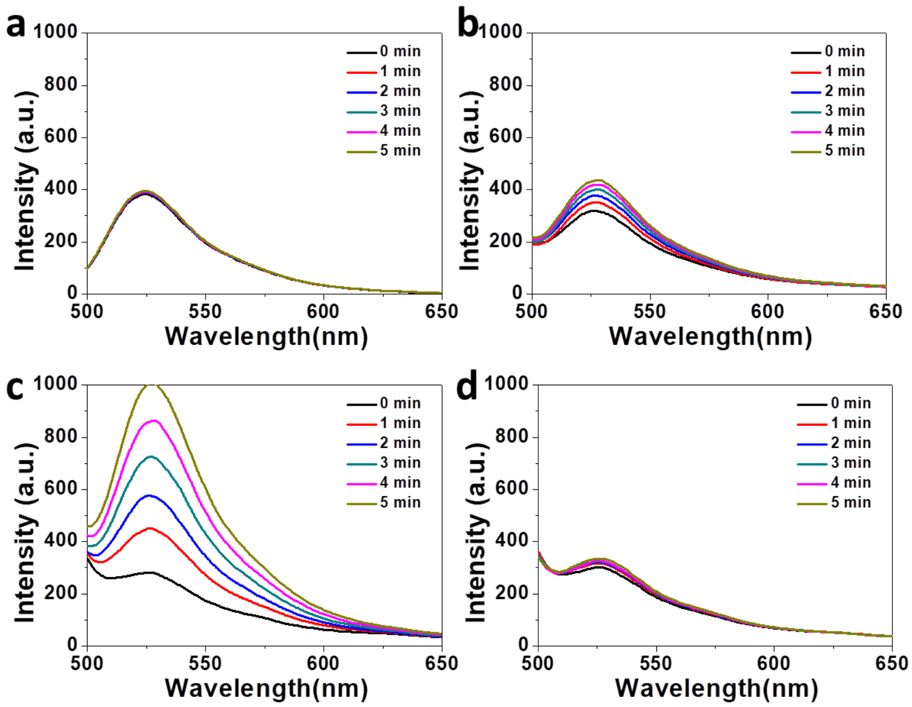

Supplementary Figure 13. Generation of R123 *via* generated $\cdot O_2^-$. **a** 658 nm laser, **b** FCL-PEG NSs, **c** FCL-PEG NSs + 658 nm, and **d** FCL-PEG NSs + 658 nm + Vc. The concentration of FCL-PEG NSs was 0.2 mg/mL. The power density of the 658 nm laser was 0.5 W/cm^2.

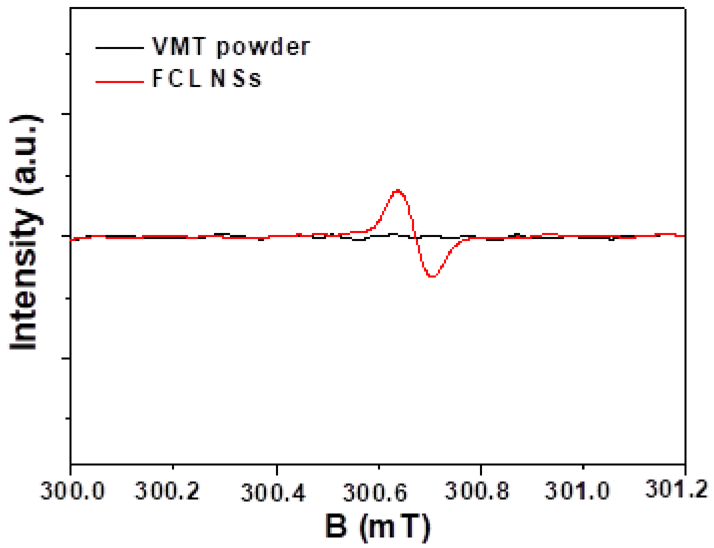

Supplementary Figure 14. EPR spectra of VMT powder and FCL NSs.

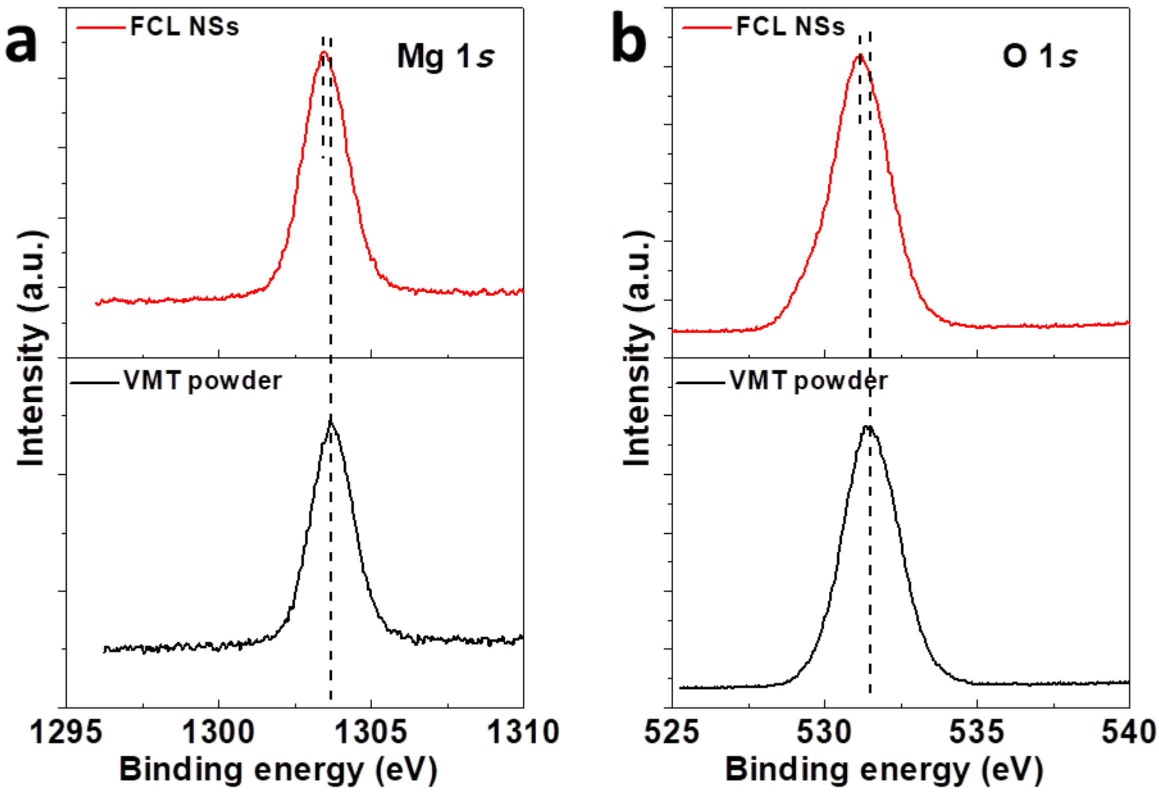

Supplementary Figure 15. High-resolution XPS spectra of VMT powder and FCL NSs. **a** Mg 1*s*, and **b** O 1*s*.

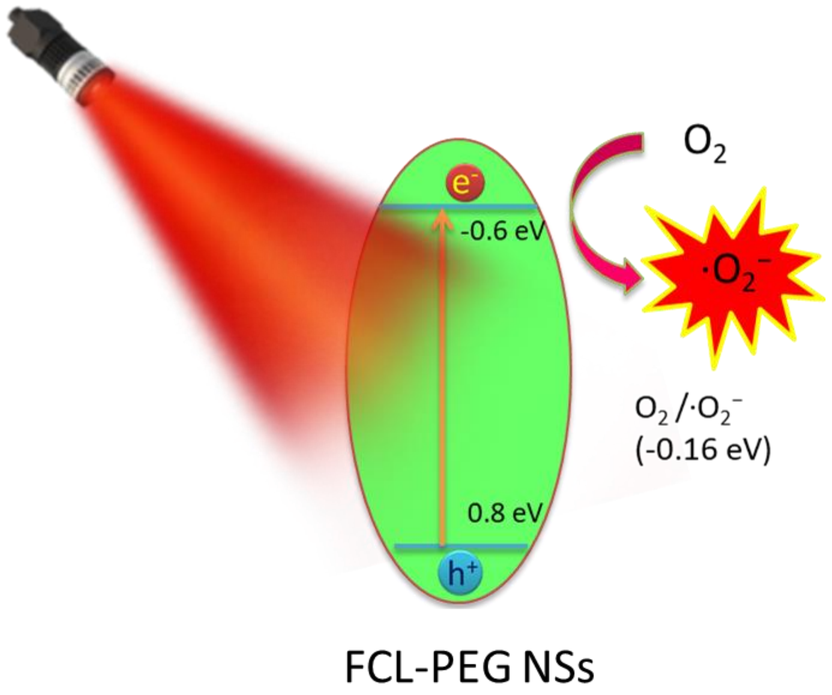

Supplementary Figure 16. Schematic illustration of FCL-PEG NSs-based PDT.

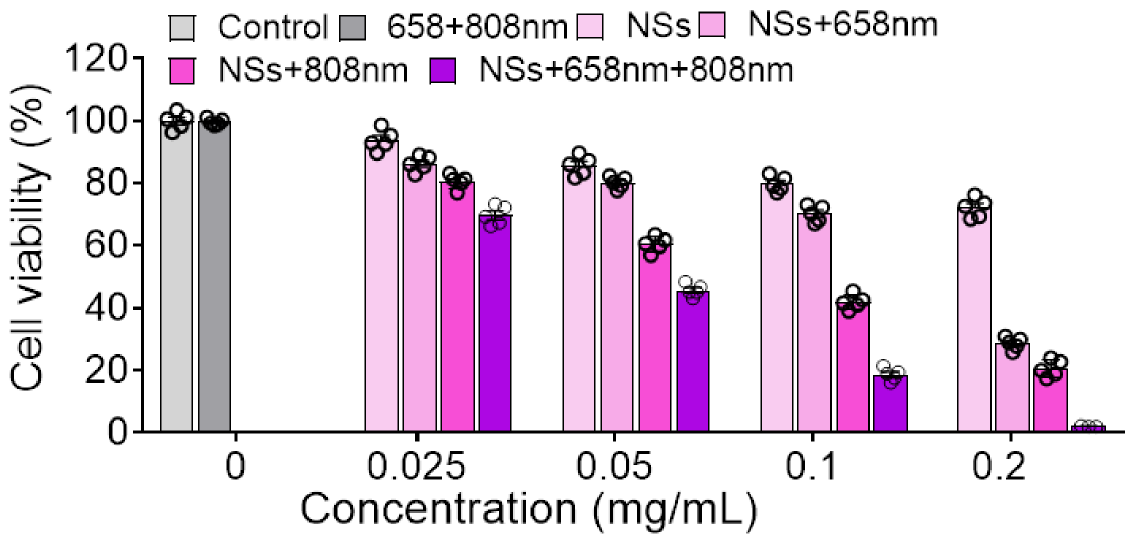

Supplementary Figure 17. Antitumor effect of FCL-PEG NSs at different concentrations on A549 cells. The data show mean ± s.d., $n = 5$ biologically independent cells.

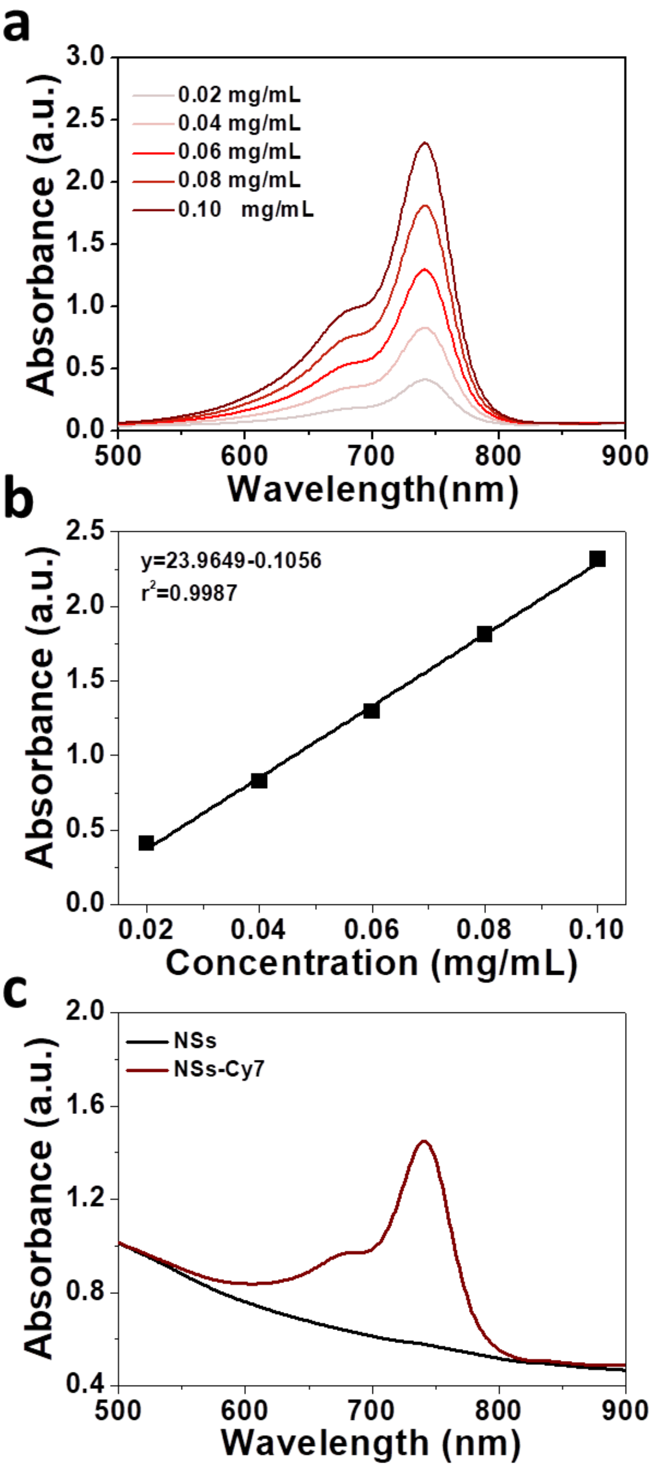

Supplementary Figure 18. Characterization of FCL-PEG-Cy7 NSs via absorbance. **a** Absorbance of free Cy7-PEG-NH_2. **b** Normalized absorbance intensity of Cy7-PEG-NH_2 at different concentrations for λ=740 nm. **c** Absorbance of FCL-PEG NSs and FCL-PEG-Cy7 NSs.

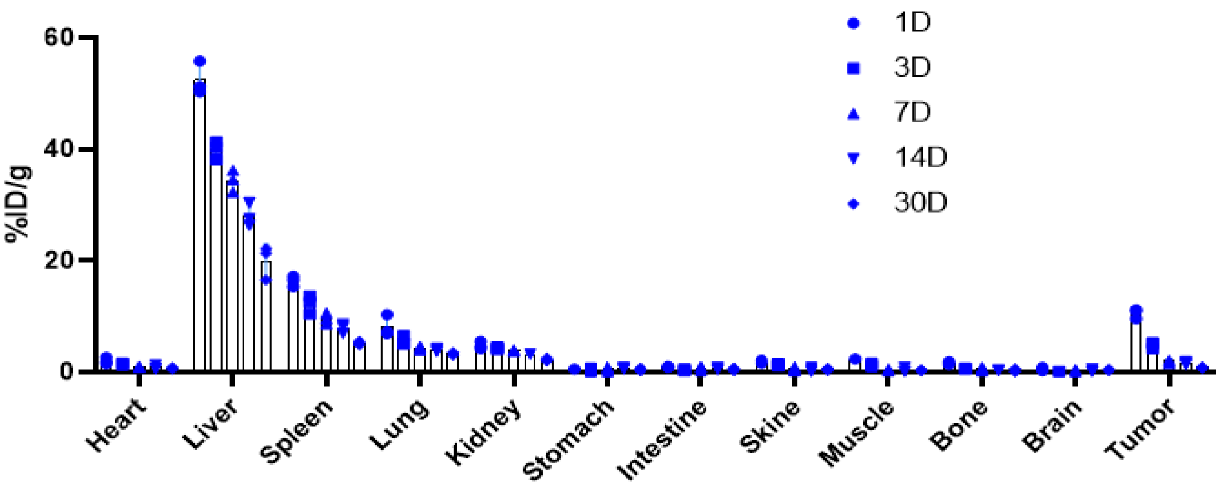

Supplementary Figure 19. Biodistribution of FCL-PEG NSs in HepG2 tumor-bearing mice by ICP measurement. The data show mean ± s.d., *n* = 3 biologically independent mice.

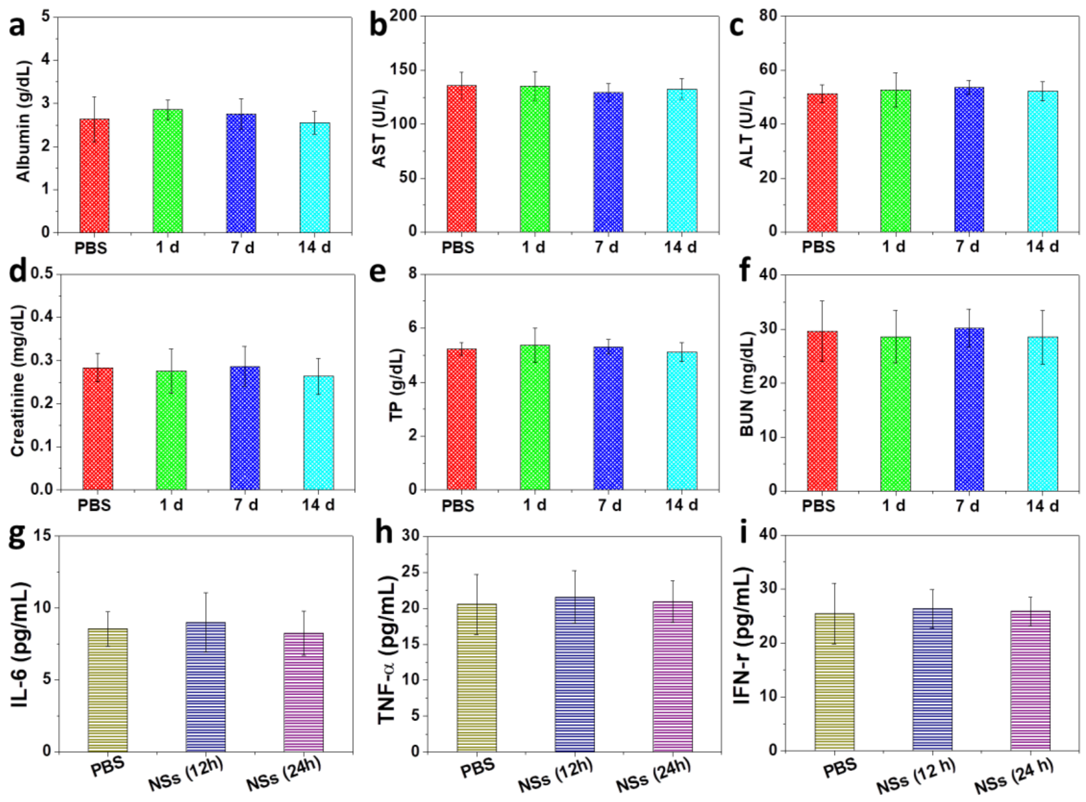

Supplementary Figure 20. Hematology assay and immune analysis. The detection of **a** albumin, **b** aspartate aminotransferase (AST), **c** alanine aminotransferase (ALT), **d** creatinine, **e** total protein (TP), and **f** blood urea nitrogen (BUN) at different times after intravenous injection of PBS versus FCL-PEG NSs. Serum levels of **g** interleukin 6 (IL-6), **h** tumor necrosis factor-α (TNF-α), and **i** interferon-γ (IFN-γ) in healthy mice at different times after intravenous injection of PBS versus FCL-PEG NSs. The data show mean ± s.d., *n* = 3 biologically independent mice.

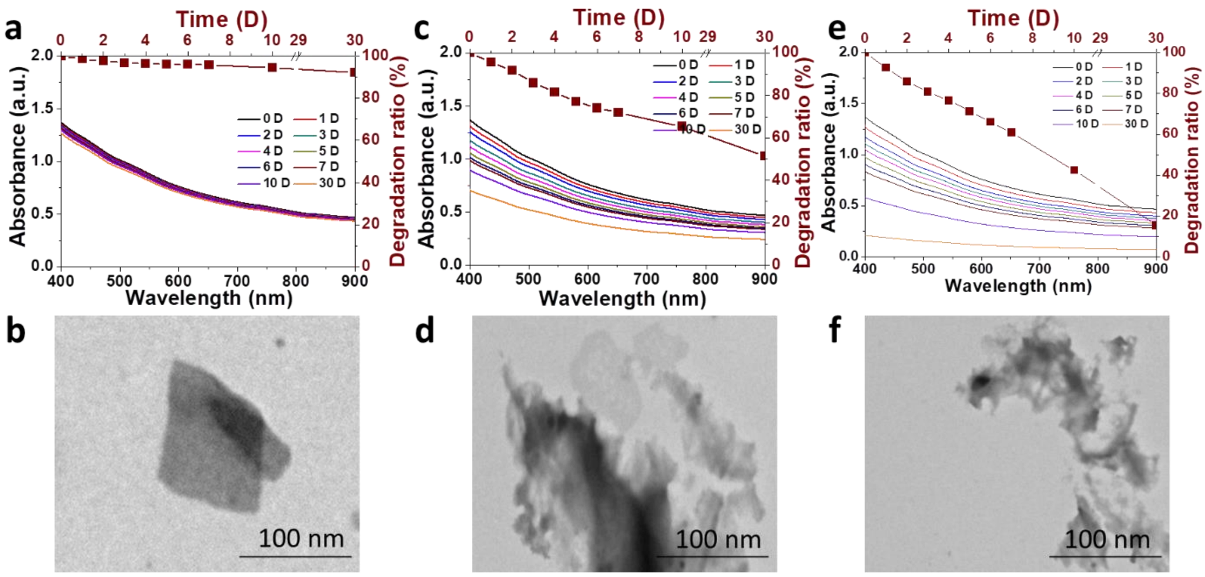

Supplementary Figure 21. Storage stability evaluation of FCL-PEG NSs over 30 days. **a** UV–vis–NIR absorbance curves and **b** TEM images of FCL-PEG NSs treated with PBS (pH 7.4) for different periods (three times each experiment was repeated independently with similar results). **c** UV–vis–NIR absorbance curves and **d** TEM images of FCL-PEG NSs treated with H_2O_2 for different periods (three times each experiment was repeated independently with similar results). **e** UV–vis–NIR absorbance curves and **f** TEM images of FCL-PEG NSs treated with PBS (pH 5.5) for different periods (three times each experiment was repeated independently with similar results).
