# Supplementary material for: Capturing functional two-dimensional nanosheets from sandwich-structure vermiculite for cancer theranostics
Source: Nat Commun. 2021 Feb 18;12:1124. doi: 10.1038/s41467-021-21436-5 (PMC7892577; doi:10.1038/s41467-021-21436-5)
Supplement: Supplementary file 2 — Reporting Summary [file 41467_2021_21436_MOESM2_ESM.pdf]

nature research

Corresponding author(s): Xiaobin Zeng, Na Kong, Xingcai Zhang, Wei Tao

Last updated by author(s): Nov 7, 2020

Reporting Summary

Nature Research wishes to improve the reproducibility of the work that we publish. This form provides structure for consistency and transparency in reporting. For further information on Nature Research policies, see our Editorial Policies and the Editorial Policy Checklist.

Statistics

For all statistical analyses, confirm that the following items are present in the figure legend, table legend, main text, or Methods section.

n/a	Confirmed	
☐	☒	The exact sample size (*n*) for each experimental group/condition, given as a discrete number and unit of measurement
☐	☒	A statement on whether measurements were taken from distinct samples or whether the same sample was measured repeatedly
☐	☒	The statistical test(s) used AND whether they are one- or two-sided<br>*Only common tests should be described solely by name; describe more complex techniques in the Methods section.*
☒	☐	A description of all covariates tested
☒	☐	A description of any assumptions or corrections, such as tests of normality and adjustment for multiple comparisons
☐	☒	A full description of the statistical parameters including central tendency (e.g. means) or other basic estimates (e.g. regression coefficient) AND variation (e.g. standard deviation) or associated estimates of uncertainty (e.g. confidence intervals)
☐	☒	For null hypothesis testing, the test statistic (e.g. *F*, *t*, *r*) with confidence intervals, effect sizes, degrees of freedom and *P* value noted<br>*Give P values as exact values whenever suitable.*
☒	☐	For Bayesian analysis, information on the choice of priors and Markov chain Monte Carlo settings
☐	☒	For hierarchical and complex designs, identification of the appropriate level for tests and full reporting of outcomes
☒	☐	Estimates of effect sizes (e.g. Cohen's *d*, Pearson's *r*), indicating how they were calculated

Our web collection on statistics for biologists contains articles on many of the points above.

Software and code

Policy information about availability of computer code

Data collection	BD FACSCalibur was used to collect and Flowjo (7.6) was used to analyze flow cytometry data. TEM data was collected using JEM-2100UHR (JEOL, Japan). AFM data was collected using atomic-force microscopy (FASTSCANBIO, Germany). EDX mapping data was collected via energy-dispersive X-ray spectroscopy (Inca X-MAX, Oxford, UK). The hydrodynamic size was measured using a Malvern Zetasizer Nano-ZS (ZEN3600). XPS data was recorded on X-ray photoelectron spectroscopy (ESCALAB 250Xi, Japan). UV-Vis-NIR data were measured using the Agilent Cary 8454 UV-Visible spectrophotometer. The absorption of FCL NSs and VMT powder was detected by solid UV-vis-NIR spectrophotometer (Hitachi, UH4150, Japan). EPR data was acquired in a Bruker EMXPlus-10/12 system. The infrared thermographic and temperatures of tumor/cell were recorded by a FLIR E6 infrared camera (Arlington, VA). FL images were collected using Maestro2 In-Vivo Imaging System. PA images were obtained by the Vevo LAZR photoacoustic imaging system (Visual-Sonics Co.). Fluorescent cell imaging was performed on KEYENCE BA-X700 (all-in-one Fluorescence Microscope). MTT data was collected using Bio-Rad Mode 680 microplate reader.
Data analysis	Analysis of flow cytometry data was performed with Flowjo v7.6 software. Graph Pad Prism (5.0a or 8.0) and Origin 9.0 were used for data statistics and statistical significance calculation. Microsoft Excel was used for biodistribution and tumor size analysis. FL and PA images were analyzed using Image J. Zetasizer Nano software v3.30 for analyses of particle size. TEM data was analyzed using Gatan-DigitalMicrograph-3.9.

For manuscripts utilizing custom algorithms or software that are central to the research but not yet described in published literature, software must be made available to editors and reviewers. We strongly encourage code deposition in a community repository (e.g. GitHub). See the Nature Research guidelines for submitting code & software for further information.

Data

Policy information about availability of data

All manuscripts must include a data availability statement. This statement should provide the following information, where applicable:

- Accession codes, unique identifiers, or web links for publicly available datasets
- A list of figures that have associated raw data
- A description of any restrictions on data availability

Provide your data availability statement here.

Field-specific reporting

Please select the one below that is the best fit for your research. If you are not sure, read the appropriate sections before making your selection.

☒ Life sciences ☐ Behavioural & social sciences ☐ Ecological, evolutionary & environmental sciences

For a reference copy of the document with all sections, see nature.com/documents/nr-reporting-summary-flat.pdf

Life sciences study design

All studies must disclose on these points even when the disclosure is negative.

Sample size	No statistical methods were used to predetermine the sample sizes. The number of animals in each group was determined according to previous studies cited in our manuscript. The size of each sample is in close agreement with those studies already published and with the need for statistical analysis to discuss the degree of differences and measure the variability of these in vivo data.
Data exclusions	No data were excluded from the analysis.
Replication	All the experimental findings were replicated with the number of replicates, animals and variation shown by n and SD. All experimental findings were replicated successfully using biological replicates on different days.
Randomization	FL and PA imaging study: animals were distributed randomly into different groups. Therapeutics study: animals were inoculate with tumor cell suspensions and after randomly distributed into the control and experimental groups. Each specific treatment was administrated to animals according to established schedules and regimens.
Blinding	Investigators were blinded when grouping tumor bearing mice, measuring tumor size, performing biodistribution and imaging study. Investigators for TEM and AFM characterization are blinded to the samples. During the experiments designed to evaluate anti-tumor efficacy, animals were inoculated with tumor cells and randomly divided into control and treatment groups. The investigators were not blinded during these pre-clinical proof-of-concept studies based on combinatorial schemes.

Reporting for specific materials, systems and methods

We require information from authors about some types of materials, experimental systems and methods used in many studies. Here, indicate whether each material, system or method listed is relevant to your study. If you are not sure if a list item applies to your research, read the appropriate section before selecting a response.

Materials & experimental systems

n/a	Involved in the study	
☒	☐	Antibodies
☐	☒	Eukaryotic cell lines
☒	☐	Palaeontology and archaeology
☐	☒	Animals and other organisms
☒	☐	Human research participants
☒	☐	Clinical data
☒	☐	Dual use research of concern

Methods

n/a	Involved in the study	
☒	☐	ChIP-seq
☐	☒	Flow cytometry
☒	☐	MRI-based neuroimaging

Eukaryotic cell lines

Policy information about cell lines

Cell line source(s)	Human lung fibroblasts (CCD-25Lu), human embryonic kidney cells (HEK 293), human liver epithelial cells (THLE-3), human hepatoma carcinoma cells (HepG2), and human lung cancer cells (A549) were obtained from the American Type Culture Collections (ATCC).
Authentication	ATCC used morphology, karyotyping, and PCR based approaches to confirm the identity of human cell lines and to rule out both intra- and interspecies contamination. Also, the cell line were frequently checked by their morphological features.
Mycoplasma contamination	All cells were negative for mycoplasma.
Commonly misidentified lines (See ICLAC register)	No commonly misidentified cell line were used.

Animals and other organisms

Policy information about studies involving animals; ARRIVE guidelines recommended for reporting animal research

Laboratory animals	6 to 8-week-old male Balb/c nude mice and male C57BL/6 mice (purchased from Guangdong Medical Laboratory Animal Center, Guangzhou, China) were used in this study.
Wild animals	No wild animals were used in this study.
Field-collected samples	This study did not involve samples collected from the fields.
Ethics oversight	All animals received humane care, and the Animal Ethics Committee of the National Institute of Radiological Sciences approved all the animal experiments. All experiments were carried out according to the recommendations of the Committee for the Care and Use of Laboratory Animals, Shenzhen People's Hospital.

Note that full information on the approval of the study protocol must also be provided in the manuscript.

Flow Cytometry

Plots

Confirm that:

- [x] The axis labels state the marker and fluorochrome used (e.g. CD4-FITC).
- [x] The axis scales are clearly visible. Include numbers along axes only for bottom left plot of group (a 'group' is an analysis of identical markers).
- [ ] All plots are contour plots with outliers or pseudocolor plots.
- [x] A numerical value for number of cells or percentage (with statistics) is provided.

Methodology

Sample preparation	Single cell suspensions from cultured cells were used for flow cytometry tests.
Instrument	BD FACSCalibur
Software	Flowjo v7.6
Cell population abundance	Relevant cell fractions were above 90% for all samples.
Gating strategy	Generally, cells were first gated on FSC/SCC. Singlet cells were usually gated using FSC-H and FSC-A. Debris were removed by thresholding.

- [ ] Tick this box to confirm that a figure exemplifying the gating strategy is provided in the Supplementary Information.
